# *In Vitro* Bone Cell Models: Impact of Fluid Shear Stress on Bone Formation

**DOI:** 10.3389/fbioe.2016.00087

**Published:** 2016-11-15

**Authors:** Claudia Wittkowske, Gwendolen C. Reilly, Damien Lacroix, Cecile M. Perrault

**Affiliations:** ^1^Department of Mechanical Engineering, University of Sheffield, Sheffield, UK; ^2^INSIGNEO Institute for in silico Medicine, University of Sheffield, Sheffield, UK; ^3^Department of Material Science, University of Sheffield, Sheffield, UK

**Keywords:** bone remodeling, collagen orientation, osteoblast, osteocyte, fluid shear stress

## Abstract

This review describes the role of bone cells and their surrounding matrix in maintaining bone strength through the process of bone remodeling. Subsequently, this work focusses on how bone formation is guided by mechanical forces and fluid shear stress in particular. It has been demonstrated that mechanical stimulation is an important regulator of bone metabolism. Shear stress generated by interstitial fluid flow in the lacunar-canalicular network influences maintenance and healing of bone tissue. Fluid flow is primarily caused by compressive loading of bone as a result of physical activity. Changes in loading, e.g., due to extended periods of bed rest or microgravity in space are associated with altered bone remodeling and formation *in vivo*. *In vitro*, it has been reported that bone cells respond to fluid shear stress by releasing osteogenic signaling factors, such as nitric oxide, and prostaglandins. This work focusses on the application of *in vitro* models to study the effects of fluid flow on bone cell signaling, collagen deposition, and matrix mineralization. Particular attention is given to *in vitro* set-ups, which allow long-term cell culture and the application of low fluid shear stress. In addition, this review explores what mechanisms influence the orientation of collagen fibers, which determine the anisotropic properties of bone. A better understanding of these mechanisms could facilitate the design of improved tissue-engineered bone implants or more effective bone disease models.

## 1 Bone Physiology

Bone is a highly specialized, rigid tissue which provides structural support for the body, allows movement through muscle attachment sites, protects organs, and serves as calcium and growth factor storage (Clarke, 2008). Bone has the power to regenerate and repair constantly throughout the entire life. This process, referred to as bone remodeling, involves different cell types and can be initiated in response to changes in biomechanical loading or to replace old, microdamaged bone with new, mechanically stronger bone (Kini and Nandeesh, 2012).

### 1.1 Bone Remodeling

**Figure 1:**
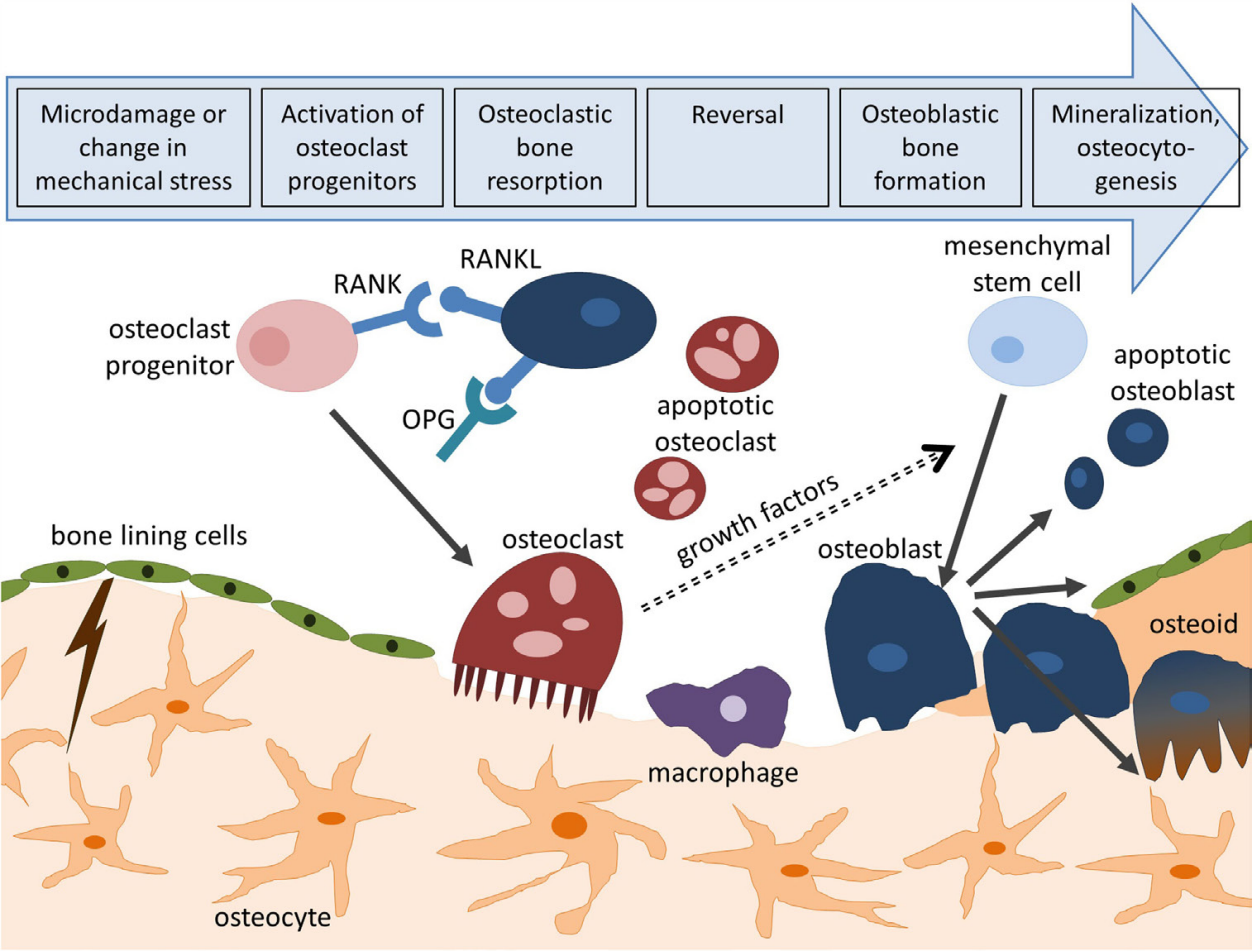
**Bone remodeling cycle**. Bone remodeling is initiated by microcracks or changes in mechanical loading and consists of four consecutive steps: activation, resorption, reversal, and formation. Activation of osteoclasts is controlled through the RANK/RANKL/OPG pathway. Following bone deposition, osteoblasts can differentiate to osteocytes (osteocytogenesis), turn to bone-lining cells, or enter apoptosis.

Bone remodeling is an essential process in maintaining bone strength and mineral homeostasis. Remodeling allows the repair of old and damaged bone and adjustment of the bone’s architecture to changes in external loading. Specialized cells, namely osteoclasts which remove mineralized matrix and osteoblasts which deposit new bone matrix, work together during this process. Their collaboration is tightly controlled through biochemical pathways (Hadjidakis and Androulakis, 2006). For example, the release receptor activator of nuclear factor kappa-B ligand (RANKL) by osteoblasts induces osteoclast activation through binding to RANK receptors on the surface of osteoclast precursors. This process can be inhibited by osteoprotegerin (OPG) which competitively binds to RANKL (Boyce and Xing, 2008). The remodeling cycle (Figure 1) is composed of four consecutive phases (Clarke, 2008):

- *Activation:* hormonal or physical stimuli recruit mononuclear pre-osteoclasts from the circulation to the bone remodeling site. Following attachment to the bone surface, cells fuse to multinucleated osteoclasts.
- *Resorption:* osteoclasts initiate resorption of organic and mineral bone components which takes between 2 and 4 weeks. Osteoclasts form characteristic Howship’s lacunae in trabecular bone and a cutting cone in cortical bone. After these cavities reach a certain size, apoptosis of osteoclasts terminates bone resorption (Sikavitsas et al., 2001).
- *Reversal:* the resorbed surface is smoothed by mononuclear macrophage-like cells and prepared for matrix deposition.
- *Formation:* osteoblasts lay down new bone by secreting a collagen matrix and controlling its mineralization. Throughout this process, some osteoblasts become buried within the matrix and differentiate to osteocytes which reside in the fully mineralized lacunar-canalicular system (LCS). After 4–6 months, this phase is completed and osteoblasts either turn into bone-lining cells or enter apoptosis.

In cortical bone, a remodeling rate of 2–3% per year is sufficient to maintain bone strength. Trabecular bone presents a higher turnover rate, indicating the importance of bone remodeling for calcium and phosphorus metabolism (Clarke, 2008).

### 1.2 Bone Cells

Bone cells work together in a coordinated way during bone remodeling by maintaining a balance between osteoblasts depositing new bone tissue, osteoclasts breaking down bone matrix, and osteocytes orchestrating the activity of osteoblasts and osteoclasts as a response to mechanical loading (Hadjidakis and Androulakis, 2006; Bonewald and Johnson, 2008).

#### 1.2.1 Osteoblasts

Osteoblasts are bone-forming cells which are derived from mesenchymal stem cells (MSC) (Caplan, 1991). MSCs differentiate into osteoblasts under the appropriate stimuli, but they can also turn into cartilage, muscle, tendon, and fat cells (Caplan and Bruder, 2001). The osteoblast differentiation and maturation process is governed by both mechanical and biochemical pathways. For example, Runt-related transcription factor 2 (Runx2) is essential in preosteoblast development where it activates osteoblast-specific genes, including osteopontin, type I collagen, osteocalcin, and alkaline phosphatase (ALP) (Ducy et al., 1997; Xu et al., 2015). Mature osteoblast differentiation is controlled by the Wnt signaling pathway, which is activated either by hormones or mechanically (Westendorf et al., 2004).

The morphology of preosteoblasts is very similar to fibroblasts; however, the latter are not able to produce a mineralized matrix. Mature osteoblasts are typically cuboidal in shape (Franz-Odendaal et al., 2006). Osteoblasts directly regulate bone matrix synthesis and mineralization by their own secretion mechanism. Bone resorption is indirectly controlled by osteoblasts through paracrine factors acting on osteoclasts. For example, the release of receptor activator of RANKL initiates bone resorption through binding to RANK receptors on the surface of osteoclast precursors (Boyce and Xing, 2008).

The average life-span of osteoblasts ranges from a few days to about 100 days (Rosenberg et al., 2012). At the end of their life, osteoblasts can either (1) become embedded in newly formed bone matrix and differentiate to osteocytes, (2) transform into inactive bone-lining cells which protect inactive bone surfaces, or (3) initiate apoptosis (Manolagas, 2000).

#### 1.2.2 Osteocytes

Osteocytes are terminally differentiated osteoblasts which became trapped within newly deposited bone matrix (Franz-Odendaal et al., 2006). Although osteoblast and osteocytes have the same origin, they significantly differ in morphology and function. During osteocytogenesis, i.e., differentiation from osteoblasts to osteocytes, the cell body size decreases and cell processes start to radiate toward the mineralizing matrix which may be controlled by E11/gp38, a marker for early osteocytes (Schulze et al., 1999). After the transition, gene expression of ALP, type I collagen, and bone morphogenetic protein 2 (BMP-2) are reduced. Other proteins, including osteocalcin, E11/gp38, sclerostin (Sost), and dentin matrix protein 1 (DMP-1) are upregulated or introduced (Mullen et al., 2013).

There is little knowledge about the cues which regulate osteocytogenesis (Dallas et al., 2013). The mechanical properties of the deposited osteoid, which is softer compared to mineralized bone tissue, might guide differentiation (Mullen et al., 2013). In addition, mineralization of the osteoid and hypoxic conditions might also be a driver for osteocyte formation (Irie et al., 2008; Prideaux et al., 2012).

Research in osteocytes has gained interest in recent years, since they are no longer seen as the “passive place holder in bone,” but as cells with very different functions (Bonewald, 2011). Osteocytes, which are the most abundant cell type in bone (90–95% of total bone cells), are thought to respond to mechanical loading by releasing signal factors. Through these factors, they coordinate bone remodeling by regulating osteoclast and osteoblast activity (Knothe Tate et al., 2004).

#### 1.2.3 Osteoclasts

Osteoclasts are specialized cells which can resorb mineralized bone matrix by secreting acid and lytic enzymes. They are multinucleated cells derived from mononuclear precursor cells which are located in the bone marrow (Boyle et al., 2003). Their differentiation (osteoclastogenesis) is controlled by cytokines, such as RANKL and macrophage colony-stimulating factor (M-CSF), which are produced by neighboring stromal cells and osteoblasts. Differentiation of osteoclasts can be inhibited by OPG which binds RANKL with high affinity and prevents its attachment to the RANK receptor (Suda et al., 1999).

### 1.3 Bone Extracellular Matrix

The extracellular matrix (ECM) of bone is a composite material consisting of 50–70% inorganic mineral, 20–40% organic materials, less than 3% lipids, and water (Clarke, 2008). The exact composition depends on factors such as age, bone site, gender, or medical conditions including osteoporosis (Boskey, 2013).

The mineral part of bone closely resembles hydroxyapatite and provides the bone with mechanical rigidity and load-bearing strength (Boskey, 2007). This phase can be best described as a crystalline complex of calcium and phosphate which also contains impurities, such as sodium, magnesium, citrate, and fluoride (Khan et al., 2013). Elasticity and flexibility of the bone is provided by the organic components which include structural proteins, such as collagen and fibronectin (Nair et al., 2013). The organic phase is also composed of other non-collageneous matrix proteins which serve important functions controlling matrix organization and mineral deposition (Young, 2003; Boskey, 2013). For example, mineralization is likely to be controlled by the small Ca^2+^-binding protein osteocalcin. Mechanotransduction is facilitated by glycoproteins, such as osteopontin and osteonectin, which can attach to integrins on cell surfaces. Osteopontin also enables the attachment of osteoclasts to bone surfaces (Gundberg, 2003). The small amount of lipids is crucial for cell signaling and ion flow (Clarke, 2008).

#### 1.3.1 Collagen Assembly

**Figure 2:**
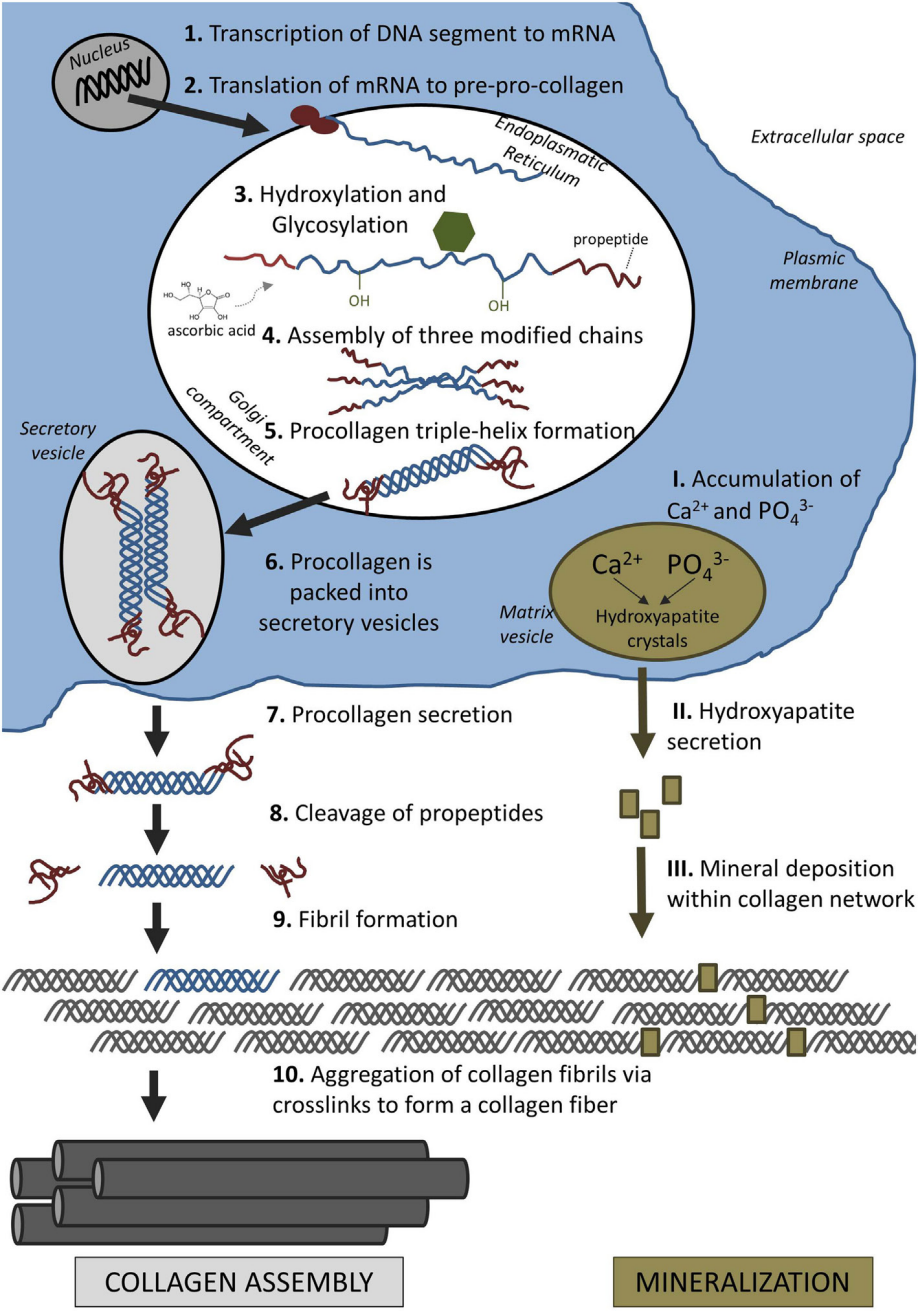
**Formation of extracellular matrix: collagen assembly and mineralization**.

The assembly of collagen fibrils is a complex process involving intracellular and extracellular steps. Collagen is first synthesized as precursor molecules (procollagen) in the intracellular space before these molecules are assembled to long fibers outside the cell (Figure 2).

Collagen formation is initiated in the nucleus of collagen-producing cells, such as osteoblasts and also fibroblasts. In the nucleus, a particular segment of deoxyribonucleic acid (DNA) is transcribed into messenger ribonucleic acid (mRNA). After the mRNA has moved out of the nucleus into the cytoplasm, it is translated into polypeptide chains, known as pre-pro-collagen. Each chain is about 300 nm in length and 1.5 nm in diameter. They are characterized by a strict pattern consisting of multiple triplet sequences of Gly–Y–Z. Glycine residues (Gly) have to be present in every third position to allow proper folding of these chains later on. Although Y and Z can be any amino acid, they are commonly proline and hydroxyproline (van der Rest and Garrone, 1991). Each chain is terminated by a few characteristic amino propeptides and carboxy propeptides on either side. These terminal propeptides are essential in preventing self-assembly of long collagen fibers within the cell.

Proline and lysine residues are then hydroxylated in the endoplasmatic reticulum (ER) which will aid cross-linking of peptide chains later. This enzymatic step requires ascorbic acid (vitamin C) as a cofactor. A lack of ascorbic acid would either result in the formation of looser collagen triple helices or prevent collagen synthesis altogether, resulting in diseases such as scurvy (Canty and Kadler, 2005). Three modified peptide chains will form a triple helix which is further stabilized by disulfide bonds. In the case of type I collagen, two *α*1 chains and one *α*2 chain assemble to form a triple helix, referred to as procollagen.

After the proteins have achieved their helical conformation, they move from the ER to the Golgi apparatus where they are packed into secretory vesicles. These carriers vary in size and morphology and have been either described as vacuoles around 500 nm in length (Leblond, 1989), or as larger tubular-saccular structures (Polishchuk et al., 2000). The vesicles move along the microtubules toward the plasma membrane where they release the procollagen in the extracellular space.

Once procollagen has been released from the cell, collagen fibers start to form directly on the cell membrane. This proximity potentially allows the cell to directly control fibrogenesis and possibly even the formation of long-range assemblies, e.g., parallel bundles in tendon and ligament or interlocking weaves in bone (Kadler et al., 2008). Fibronectin and specific cell-surface integrins, such as the collagen-binding *α*2*β*1 integrin, have been found to be essential in the organization and deposition of fibrillar collagen (McDonald et al., 1982; Li et al., 2003). Wenstrup et al. (2004) also found that small amounts of type V collagens are necessary for the induction of fibrillogenesis of collagen I fibers *in vivo*.

Collagen fibers can only form after specific enzymes remove the terminal propeptides from the procollagen which are then called tropocollagen (Prockop et al., 1998). Tropocollagen units assemble spontaneously into collagen fibers. Several hundred tropocollagen molecules line up in a characteristic “quarter staggered” array, so that the composite fiber appears as a striated pattern by electron microscopy. The striated pattern results from the longitudinal staggering of the molecules which leaves a “hole” roughly the size of one quarter of the length of tropocollagen (67 nm) between the end of one molecule and the beginning of the next (Scott, 1995). The fibers are further supported through the formation of covalent bonds. The enzyme lysyl oxidase catalyzes the formation of bonds by converting hydroxyl groups on lysines and hydroxyl lysines into aldehyde groups (Kagan and Li, 2003). Consequently, the fibers increase up to 10-fold in diameter and dramatically in length following lateral and end-to-end fusion (Birk et al., 1995).

#### 1.3.2 Collagen Orientation

**Figure 3:**
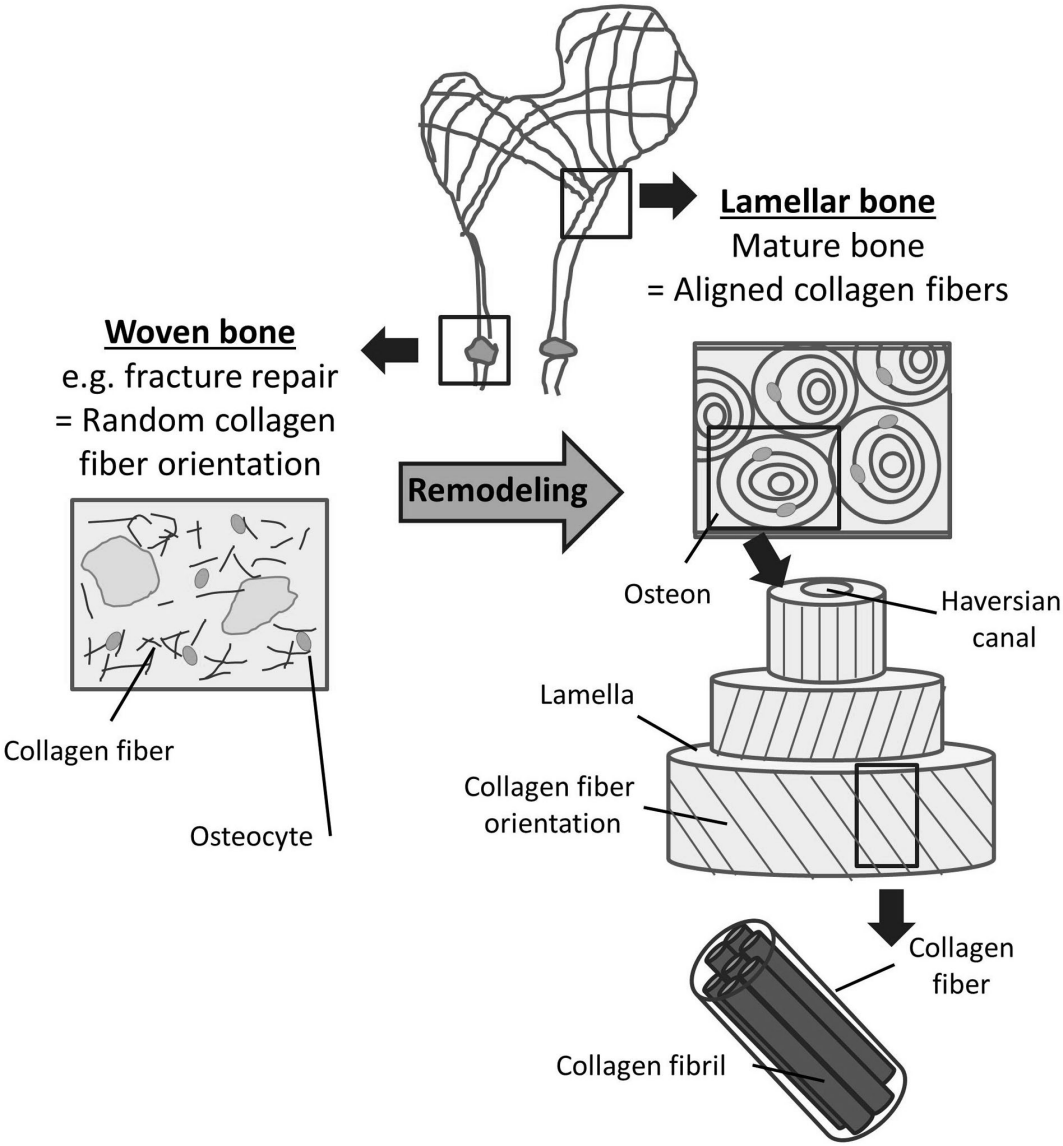
**Woven and lamellar bone**. Two types of bone can be distinguished based on the orientation of collagen fibers within the bone matrix. Woven bone (left) consists of randomly oriented collagen fibrils and exists during fetal development and fracture repair. Following bone remodeling, most mature bone can be characterized as lamellar bone (right). Lamellar bone is characterized by highly orientated collagen fibers arranged in lamellae and osteons. The alternating orientation of the collagen lamellae results in significantly higher strength of lamellar bone compared to woven bone.

Two types of bone can be distinguished based on the orientation of collagen fibers within the bone matrix: (1) woven bone which consists of randomly oriented collagen fibrils and (2) lamellar bone which is characterized by highly orientated collagen fibers (Kini and Nandeesh, 2012). Collagen fibers in lamellar bone are arranged in arrays of parallel fibers, which successively change orientation to form a “twisted plywood-like” structure (Weiner et al., 1997). The alternating orientation of collagen arrays results in significantly higher strength of lamellar bone compared with woven bone (Clarke, 2008). This plywood structure can be found in the cylindrical osteons which are the primary building blocks of compact bone. Osteons are made up of several concentric lamellae which are arranged in different orientations around the Haversian channel containing the blood and nerve supply (Figure 3). The long axis of an osteon is usually parallel to the long axis of the bone, and the dominant collagen fiber orientation commonly follows the direction of load (Martin and Boardman, 1993; Hert et al., 1994; Seto et al., 2008). Furthermore, longitudinal collagen fibers are primarily present in regions supporting tensile loads, while regions under compressive loading are composed of transverse fibers (Riggs et al., 1993; Martin et al., 1996).

Besides the spatial orientation of collagen fibers, mechanical properties of bone are also determined by several other factors which include total bone mass and material properties of bone mineral (Viguet-Carrin et al., 2006). While the latter is more important with respect to bone stiffness and yield strength, the collagen network appears to have a greater impact on post-yield properties, including ultimate strength and toughness of bone (Garnero, 2015). Even though the dependence of fiber orientation on bone strength is well accepted, it remains difficult to estimate exactly the effect collagen orientation has on mechanical properties of bone (Viguet-Carrin et al., 2006).

The mechanisms which guide the spatial arrangement of collagen fibers have been a matter of research for more than 40 years. In 1975, Jones et al. (1975) observed that collagen fibers were oriented in the same direction as osteoblasts producing the collagen, but they could not determine whether the cells controlled the orientation of the collagen fibers or vice versa. The central question since then has been revolving around whether the 3D organization of collagen fibrils is a result of self-assembly (Giraud-Guille et al., 2008) or cell activity (Matsugaki et al., 2015b). It is generally accepted that collagen fiber formation on the nanometer length scale is driven by self-assembly (Kadler et al., 2008), while, on a macro scale, there is clear evidence of collagen fiber orientation following external loading patterns as described above (Martin et al., 1996; Puustjärvi et al., 1999). As a result, research focusses on determining the mechanisms which regulate collagen fibril assembly on intermediate length scales and whether cells influence this process (Kerschnitzki et al., 2011).

Mechanically weaker woven bone is produced in situations where no bone matrix is present, i.e., during bone formation in the fetus and newborn or in early phases of bone repair following fractures and osteotomies. Under such conditions, mesenchymal osteoblasts secret collagen fibers rapidly and randomly in all directions (Shapiro, 1988, 2008). However, most bone in a healthy adult which is formed as a result of the remodeling process described above is composed of highly orientated, lamellar bone (Figure 3). It is thought that the production of parallel collagen fibers requires collective, organized action of bone-producing cells (Kerschnitzki et al., 2011).

Weakly organized woven bone or pre-existing old bone matrix is believed to act as trigger for cells enabling lamellar bone formation. In particular, the nanofibrillar topography of bone appears to be a powerful substrate-specific cue for cell and collagen alignment. *In vitro* studies have shown that osteoblasts align uniformly along the direction of grooves in the micrometer (Wang et al., 2000, 2003) and nanometer range (Zhu et al., 2005; Yang et al., 2009; Lamers et al., 2010). Highly oriented natural and artificial collagen fiber matrices can also act as scaffold inducing alignment of osteoblasts (Delaine-Smith, 2013; Matsugaki et al., 2015b). These studies further show that newly secreted collagen fibers and apatite crystals follow the cell direction. In contrast, Matsugaki et al. (2015a) recently cultured osteoblasts on nanogrooved biomedical alloys. However, they observed a mismatch between cell orientation following the direction of the grooves and collagen matrix and apatite crystals orientation which aligned perpendicular to the cell direction. The authors explained this “abnormal” orientation with a yet-to-define impact of pattern spacing and the potential existence of a threshold for parallel or perpendicular organization of bone matrix produced by osteoblasts.

Mechanical stimulation might be another trigger coordinating 3D matrix arrangement. For example, osteoblasts collectively change orientation under cyclic stretching probably to minimize strain applied to them and secrete better organized collagen bundles (Matsugaki et al., 2013). The influence of mechanical forces on collagen fiber alignment has been extensively researched in soft tissues where fibroblasts deposit an unmineralized collagen matrix. For example, interstitial fluid flow might act as mechanical stimuli for fibroblast-mediated collagen alignment. Ng et al. (2005) observed perpendicular cell and collagen fiber alignment relative to the direction of fluid flow in a 3D collagen model. They explained the perpendicular fiber alignment with a reduction in shear and drag forces affecting the fibroblasts (Pedersen et al., 2010).

Most of the hierarchical fiber assembly is believed to take place extracellularly. However, some suggest a fiber pre-orientation already inside the transport vesicles before the procollagen is released from the cell (Leblond, 1989). In addition, cell-surface “fibropositor” structures have been described in embryonic tendons indicating that there might be mechanisms in place which determine fiber orientation before the extracellular release of procollagens (Holmes et al., 1998; Canty and Kadler, 2005). Importantly, to date, this feature appears to be specific to tendon development only and has not been found in bone-producing osteoblasts.

In bone, the mechanisms involved in the alignment of collagen fibers by osteoblasts are not well understood yet (Matsugaki et al., 2013). In soft tissues, traction forces exerted by fibroblasts are thought to be the main driver in remodeling ECM (Harris et al., 1981; Feng et al., 2014). For example, contraction of myofibroblasts and the transmission of these forces to the collagen network are considered a crucial step in wound healing (Tomasek et al., 2002). Traction forces applied by osteoblasts might be able to orient fibers in a similar way as observed with fibroblasts (Curtze et al., 2004). Using traction force microscopy, Poellmann et al. (2015) demonstrated that differentiated, collagen-producing osteoblasts exert higher traction forces compared with preosteoblasts found in the marrow which are not yet involved in collagen secretion. In addition, time-lapse images suggest that traction forces combined with cell motility enables osteoblasts to actively reorganize the ECM by moving “packets” of fibriliar material around (Dallas, 2006). Traction forces induced by osteoblasts might also create strains within the osteoid layer which initiate collagen fiber alignment through degradation of unstrained collagen fibers (Flynn et al., 2010; Heck et al., 2015). Furthermore, interaction between fibronectin, integrins, and other collagens, such as collagen V, may also play a role in the collagen fiber orientation process *in vivo* (Kadler et al., 2008).

#### 1.3.3 Mineralization

The organic collagen matrix in bone is strengthened by a mineral phase. Similar to the deposition of the organic collagen matrix, mineralization is also a cell-controlled process. For example, osteoblasts initiate calcification at selected, non-random sites and they regulate the ion flux into the ECM (Boskey, 2007).

Mineralization occurs in two steps (Figure 2). First, hydroxyapatite crystals are formed within matrix vesicles inside the osteoblast. In the second step of mineralization, hydroxyapatite is secreted through the membrane into the ECM and deposited within the collagen fibrils (Anderson, 1995).

During the first phase, calcium ions and phosphates are accumulated in matrix vesicles. This step is controlled by calcium-binding molecules and enzymes, including ALP (Anderson, 2003). ALP is a plasma membrane-bound enzyme, which is thought to promote mineralization by increasing the local concentration of phosphate and by breaking down extracellular mineralization inhibitors, such as pyrophosphates (Golub and Boesze-Battaglia, 2007). Once the concentration of calcium ions and phosphates exceed their solubility point, hydroxyapatite is formed within the matrix vesicles (Orimo, 2010).

During the second phase of mineralization, matrix vesicle walls are broken down and hydroxyapatite crystals are secreted into the extracellular space. The organic collagen network acts as scaffold for mineral deposition and together with non-collageneous proteins defines the size and distribution of apatite crystals in the bone (Wang et al., 2012). The small crystals are first deposited at the gap zones within the quarter-staggered collagen fibrils (Katz et al., 1989). Initially, apatite crystals align their *c*-axes parallel to collagen fibers, but ultimately all available intra-fibrillar space is filled with mineral (Golub, 2009). Fluid shear stress (FSS) might also influence formation of bone apatite, since better organized apatite crystals were formed under low FSS environment (≤1 Pa) compared to higher FSS (Niu et al., 2016).

## 2 Bone Mechanotransduction

The mechanism through which cells convert mechanical stimuli into biochemical responses is called mechanotransduction (Ingber, 2006). It is widely accepted that mechanical forces influence cell behavior and play a central role both in normal tissue physiology and diseases. Endothelial cells, for example, experience one of the greatest forces within mammalian tissues (2–4 Pa) as a result of blood shear and pressure. These cells have been shown to alter their cell morphology and orientation and determine vascular physiology and pathology as a result of fluid shear stress (Davies, 1995). Other examples include the auditory system and lungs. It has been shown that mechanotransduction is fundamental for the ability to hear. Changes in sound pressure bend hair cells in the inner ear and initiate a cascade of biochemicals signals, e.g., release of Ca^2+^ (Vollrath et al., 2007; Gillespie and Müller, 2009). Lung function is also controlled by mechanical forces which include tissue strain, fluid shear stress, and compression (Schumacker, 2002).

Similarly, mechanical loading has been identified as one of the main drivers on the mass and structural adaptation of bone. In the 19th century, Julius (Wolff, 1892) postulated, in “Wolff’s law of bone transformation,” the idea that bone architecture is a result of mechanical stress and related it to a mathematical law. Although more recent research has shown that some of Wolff’s assumptions were incorrect, the general idea that mechanical forces are closely linked to bone adaptation remains (Lee and Taylor, 1999).

Several *in vivo* studies demonstrated that gravitational forces and mechanical loads generated by muscle contractions are essential for stimulating bone remodeling and maintaining optimal mechanical performance. For example, reduction in mechanical stimulation due to extended periods of bed rest (Leblanc et al., 1990), microgravity in space (Vico et al., 2000) or limb paralysis (Weinreb et al., 1989), are associated with reduced bone formation and increased bone resorption.

### 2.1 Mediators of Mechanotransduction in Bone

**Figure 4:**
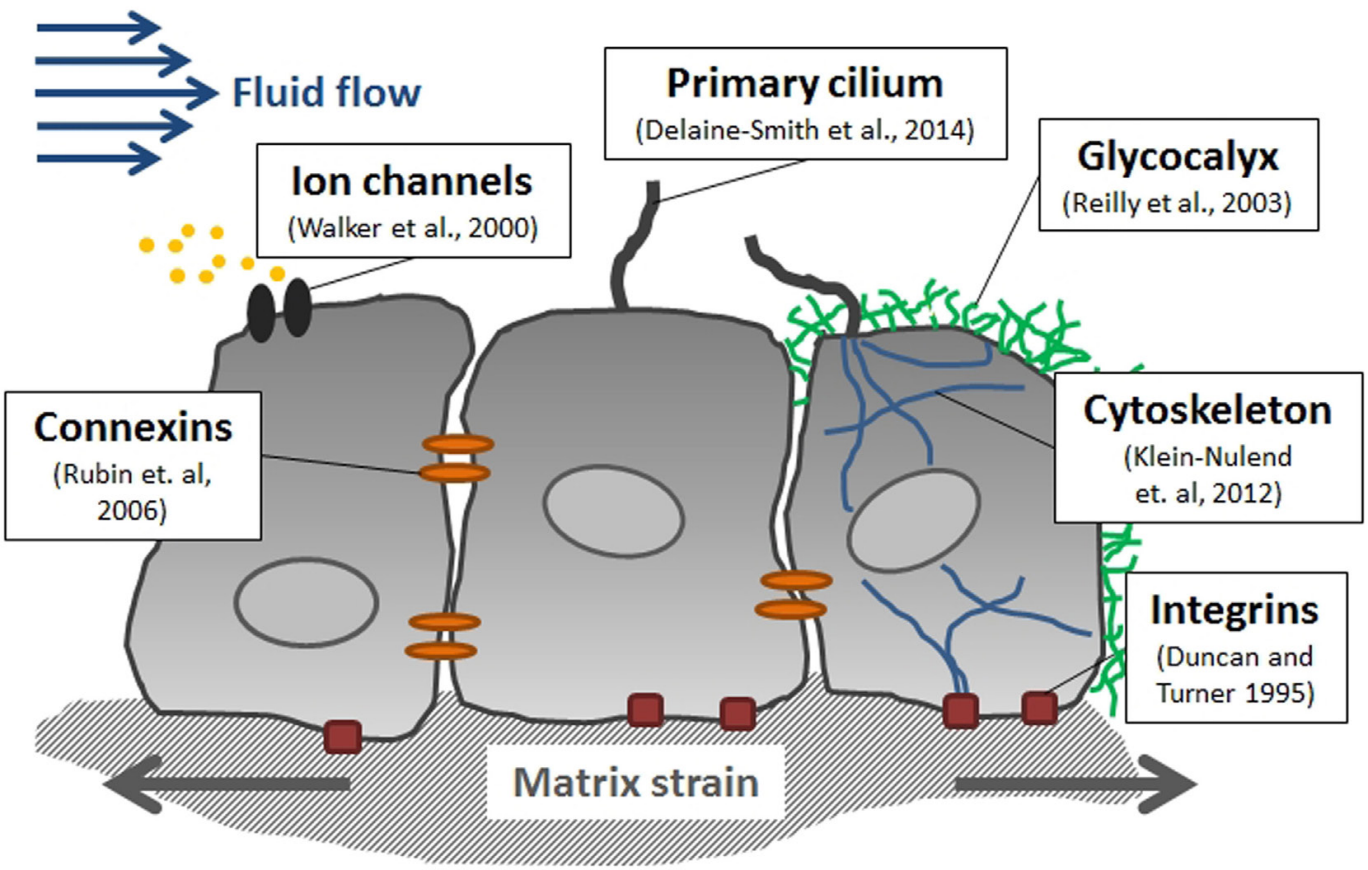
**Mediators of mechanotransduction in bone**. Several cellular structures have been identified in bone cells which contribute to sensing mechanical stimuli including fluid flow-induced shear stress and matrix strain.

Mechanical forces can be sensed and tranduced by various means at the cellular level in bone (Figure 4) (Freund et al., 2012). The matrix-integrin-cytoskeleton pathway is thought to play an important role in bone mechanotransduction, since integrins directly connect bone cells with their ECM. Integrins are membrane-bound glycoproteins which allow rapid transmission of physical stimuli from the ECM via the cytoskeleton to the nucleus, where they could initiate changes in gene expression (Duncan and Turner, 1995). The cytoskeleton itself, which is composed of actin, microtubules, and intermediate filaments, not only connects all components of the mechanosensing system but actin fibers in cell processes have also been shown to be crucial for osteocyte mechanosensing (Klein-Nulend et al., 2012). The primary cilium, which is a microtubule-based antenna-like extension, has been identified as another mechanosensor in bone cells (Malone et al., 2007; Delaine-Smith et al., 2014). The glycocalyx, which is a cellular coating rich in hyaluronic acid, might also contribute to bone cell mechanotransduction via force transmission to the cytoskeleton and integrins (Reilly et al., 2003; Burra et al., 2011). Moreover, membrane-bound proteins, such as connexins, allow exchange of molecules between adjacent cells and are, therefore, thought to be essential in osteocyte communication (Rubin et al., 2006). In addition, channels which are sensitive to fluid flow or membrane stretch can respond rapidly to mechanical stimulation by admitting or releasing ions, such as Ca^2+^ (Walker et al., 2000).

### 2.2 Mechanical Forces in Bone

Gravitational forces and muscle contractions result in small deformations of bone which generate matrix strain and interstitial fluid flow within the porous bone.

#### 2.2.1 Matrix Strain

Small deformations of mineralized bone induce tissue strain which – to some extent – may be directly sensed by bone cells. In 1987, Frost related macrostructural bone deformations to bone remodeling in his “mechanostat theory,” where he postulated that physiological strains range between 0.03 and 0.15%. Below this range, bone absorption is initiated, and between 0.15 and 0.3% bone mass is increased through bone remodeling (Frost, 1987). These thresholds have been confirmed experimentally *in vivo* (Frost, 2003; Al Nazer et al., 2012). *In vitro*, however, cells require much larger strains (1–10%) to induce osteogenic responses (Klein-Nulend et al., 1995b; You et al., 2000); strains of this magnitude would cause bone fracture *in vivo*. Therefore, cells might not directly sense bone strains as described by Frost, but rather microstructural strains near lacunae and microcracks within the bone. These strains are thought to be several times greater and might be able to stimulate osteoblasts directly (Bonivtch et al., 2007).

#### 2.2.2 Fluid Shear Stress

Interstitial fluid (ISF) is a main component of body mass (up to 20%) and is distributed throughout the ECM. It provides cells with nutrients and waste removal (Bijlani, 2004) and can also be found in cortical and cancellous bone where it fills the porosities within the tissue. The three levels of porosities in bone are: (1) the vascular porosity within the Volkmann canal and the Haversian canals (20 μm radius), (2) the lacunar-canalicular system (LCS), which are the channel structures within the mineralized bone tissue surrounding osteocytes and their processes (0.1 μm radius), and (3) tiny spaces between crystallites of the mineral hydroxapatite and collagen fibers (0.01 μm radius) (Cowin and Cardoso, 2015).

ISF flow is generally linked with lymphatic drainage, where plasma that has leaked out of the capillaries is being returned to the blood circulation (Swartz and Fleury, 2007). The hydrostatic and osmotic pressure differences between blood, interstitium, and lymphatics are considered the driving forces for the slow but constant ISF present in most soft tissues and they also affect flow in the vascular porosity and the small channels of the LCS, in particular during rest periods without physical activity (Xing et al., 2014).

**Figure 5:**
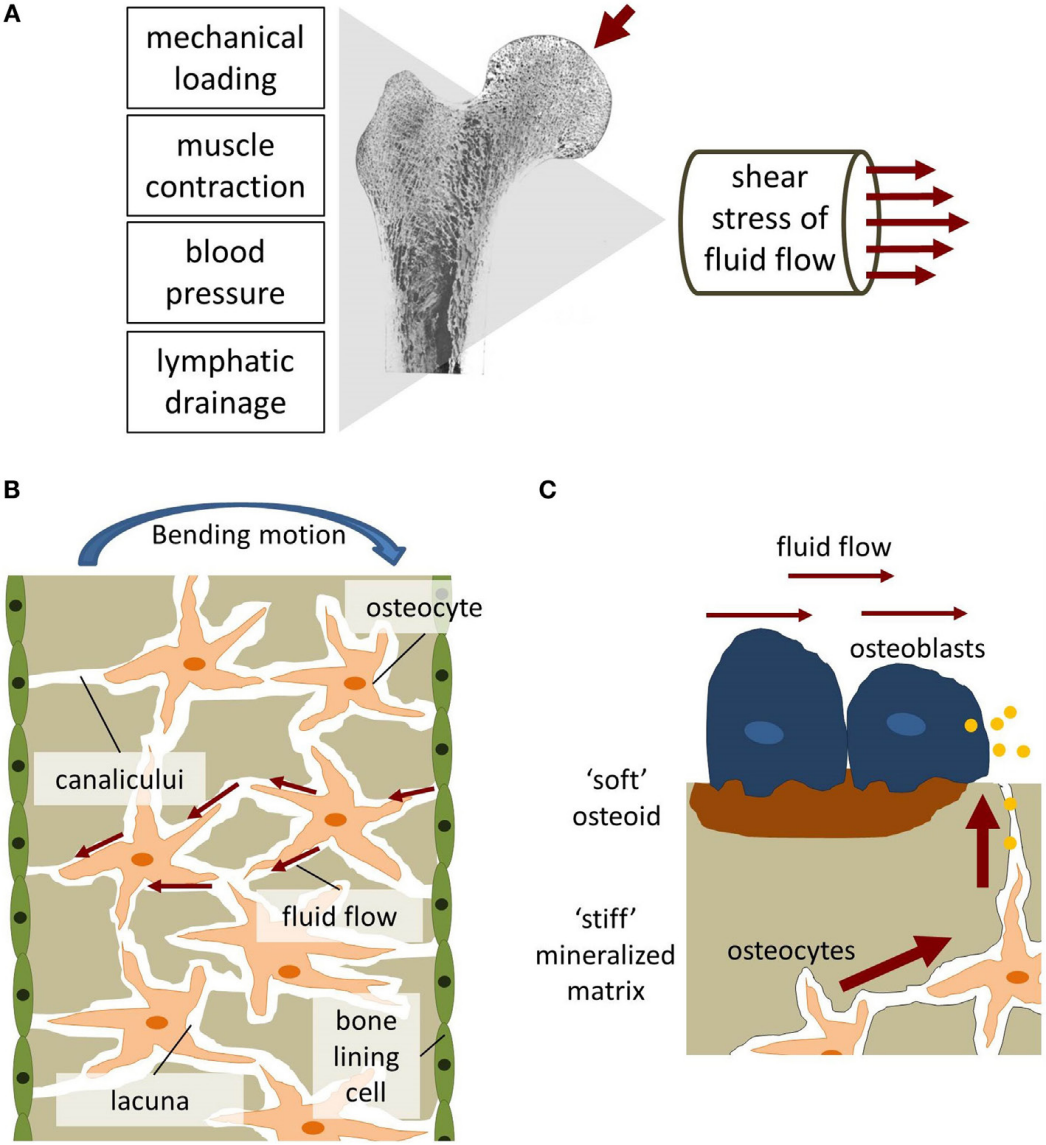
**Fluid shear stress in bone**. **(A)** Fluid flow in bone can be generated through mechanical loading, muscle contraction, blood pressure, and lymphatic drainage. **(B)** Bending of bone generates tension and compression forces which initiates interstitial fluid flow [adapted from Duncan and Turner (1995)]. **(C)** The biomechanical environment experienced by osteoblasts and osteocytes differs regarding flow velocities and matrix stiffness.

In contrast to ISF flow in most soft tissues, substantially greater flow rates can be generated in bone tissue by muscle contractions inducing blood pressure changes and mechanical loading (Figure 5A) (Burger and Klein-Nulend, 1999; Knothe Tate et al., 2000). Mechanical loading results in bending of bones and matrix deformation. Compressive stress is generated on one side of the bone and tensile stress on the other (Figure 5B). The resulting pressure gradient in the ISF is thought to drive the fluid from regions of compression to tension (Duncan and Turner, 1995). ISF has to squeeze through the narrow channels, canaliculi, which connect osteocytes residing in small spaces, lacunae, within the mineralized bone matrix. Due to the small dimensions of the channels, high wall shear stress comparable to vascular wall shear stress is generated. A numerical model by Weinbaum et al. (1994) estimated the mechanical loading-induced FSS sensed by osteocytic processes within their proteoglycan filled canaliculi to range between 0.8 and 3 Pa.

More recently, Weinbaum and colleagues put forward an alternative hypothesis which advocates fluid shear-induced strain instead of FSS as dominant mechanical stimuli in osteocytes. The group developed mathematical models, which predict strong mechanical stimulation due to fluid drag forces on actin filament bundles of the cell processes and tethering fibers. Tethering fibers anchor osteocyte cell processes to the canalicular walls and center them within the canaliculi. Fluid drag forces generate a “circumferential hoop strain” which can be compared to the force applied by pulling a ring off a finger. These forces are thought to be 1–2 magnitudes larger than whole tissue strains and several times larger than fluid shear forces on the cell surface (You et al., 2001b, 2004; Han et al., 2004). Interestingly, this hypothesis yet again puts strain (although this time it is fluid shear-induced strain) at the focus of attention.

Defining the mechanical environment of osteoblasts is even more challenging than of osteocytes. It is unlikely that the model by Weinbaum et al. (1994) can also be applied for matrix-depositing osteoblasts. Unlike osteocytes, osteoblasts are not located within tiny channels and surrounded by a stiff, calcified bone matrix. Osteoblasts can be found on the surface of soft osteoid and newly formed bone mineral at remodeling sites, i.e., in regions with bigger porosities and hence reduced fluid flow and FSS (Liegibel et al., 2004). Estimation of FSS values is further complicated due to the constant remodeling of the channel geometries which surround osteoblasts and a lack of knowledge regarding the mechanical properties of the soft osteoid. Based on experimental and computational studies, McGarry et al. (2005) hypothesized that the *in vivo* mechanical environment of osteoblasts is distinctively different to osteocytes. Bonewald and Johnson (2008) further stress that osteoblasts are unlikely to be subjected to great FSS *in vivo* and that the form and magnitude of FSS are very different from what osteocytes sense in the LCS. Therefore, even though medium to high FSS (>0.8 Pa) might be the dominant stimulus for osteocytes within the LCS and under mechanical loading during physical activity, osteoblasts might regularly encounter lower, interstitial-like FSS (Figure 5C).

#### 2.2.3 Matrix Strain or Fluid Shear Stress?

Matrix strain and fluid shear stress both cause cell deformation. However, the nature of the deformation is not the same. While strain is applied directly through the cell attachments, fluid flow is sensed through the cell membrane, first (Mullender et al., 2004). Consequently, it appears that both mechanisms excite different signaling pathways (McGarry et al., 2005). There are only few *in vitro* studies which directly compare effects of fluid flow and mechanical strain on bone cells, and, although cells often react to both stimuli, greater responses can be seen under fluid flow (Owan et al., 1997; You et al., 2000). Therefore, this review focuses on studies that investigate the direct impact of fluid shear stress on bone cell behavior.

### 2.3 *In Vitro* Models for Fluid Shear Stress Mechanotransduction

Our understanding of mechanotransduction in bone cells has been greatly enhanced by *in vitro* models (Delaine-Smith et al., 2015). *In vitro* models aim to recreate the fluid shear forces found within the bone in a controlled cell culture environment. Since the magnitude of fluid shear forces in bone is still debated, *in vitro* models prove useful for studying the impact of tightly defined types of flow, for example, by comparing unidirectional to oscillatory flow or varying the magnitudes of fluid flow.

#### 2.3.1 Parallel-Plate Flow Chamber

Fluid shear stress is commonly applied to monolayer cell cultures in a parallel-plate flow chamber (PPFC). Most designs of PPFC are modifications of the original set-up by Frangos et al. (1985), where cells were grown on a glass slide encased by a polycarbonate chamber, sealed with a rubber gasket and the fluid flow was controlled via hydrostatic pressure. Fluid flow can also be controlled by pumps generating unidirectional (Reich and Frangos, 1991; Genetos et al., 2004), pulsatile (Reich and Frangos, 1991; Hillsley and Frangos, 1997; Klein-Nulend et al., 1997; Bacabac et al., 2004), and oscillatory (Jacobs et al., 1998; Lu et al., 2012) flow profiles. Physiologically relevant wall shear stresses in the range of 0.001–3 Pa can be generated with PPFC (Yu et al., 2014). In particular, the application of very high shear rates is an advantage of PPFC compared to other systems.

Except near the inlet, flow in PPFC can be considered laminar since Reynolds numbers are generally very small. Laminar flow generates a simple flow profile resulting in a high degree of control over the flow pattern and generated shear stresses. Shear stress *τ* acting on the cells inside the channel can be calculated using the following equation:

(1) $$\tau =\frac{6\mathrm{Q\mu}}{{\mathrm{bh}}^{2}}$$

where *Q* is the flow rate, *μ* is the viscosity of the flow media, *h* is the height of the channel, and *b* is the width of the channel. In most chambers, more than 85% of the cells are exposed to a homogeneous wall shear stress for *b*/*h* > 20 (Bacabac et al., 2005). However, to ensure this uniformity, flow profiles should be verified for each channel design with numerical simulations or experimental methods, such as particle image velocimetry (PIV) (Anderson et al., 2006).

Pump-driven flow often generates high pressure at the chamber inlet, which easily causes leakages (Anderson et al., 2006). Moreover, such systems subject cells not only to FSS but also to substantial pressures. A closed loop system where fluid is simultaneously pushed and pulled can provide an alternative method and significantly reduce pressure build-up within the device (Huesa et al., 2010).

In order to perform experiments more efficiently and to test different shear rates at the same time, the basic concept of PPFC has been extended to multi-shear devices. For example, Yu et al. (2014) designed a complex microfluidic network consisting of relatively large cell culture chambers. They were able to generate different FSS on the same chip by using different widths and lengths of the inlet channels.

PPFC can also be combined with micro-patterned systems which enable cell organization. For example, Lu et al. (2012) applied micro-contact printing and self-assembled monolayer surface chemistry technologies to establish an osteocyte network on a chip.

One drawback of PPFC is that flow regimes can rarely be applied for more than 24 h. Commonly associated problems of PPFC include the formation of air bubbles within the channels which completely alter the biochemical and biomechanical environment of cells (Anderson et al., 2006).

#### 2.3.2 Multi-Well Plates on Rocking Platforms

Alternative methods for long-term culture of cells under fluid stimulation include culturing cells in multi-well plates. FSS can be applied to cell monolayers in these plates via rocking or orbital shaking platforms. Both systems have the advantage of allowing a high-throughput of samples. However, both systems are only able to generate low magnitude FSS with non-uniform patterns. The magnitude of shear stress sensed by the cells depends on several factors, most importantly their location within the well, the frequency of movement, and the amount of liquid in the well.

Rocking “see-saw” systems generate oscillatory FSS in the range of 0.001–0.25 Pa. The characteristic shear stress *τ* at the bottom of the plate can be estimated using an analytical model described by Zhou et al. (2010):

(2) $$\tau =\frac{{\pi \mu \theta }_{\max}}{2\delta^{2}T}$$

where μ is the fluid viscosity, *θ_max_* the maximal flip angle, *δ* the ratio of fluid depth to well length, and *T* the time for one cycle.

The “sea-saw” rocker system has also been characterized with a computational finite element model and validated with PIV measurements. For a rocking frequency of 0.5 Hz, a maximum shear stress of 0.22 Pa was detected near the well edge but the time-averaged shear stress along the majority of the plate center line was much lower with 0.033 Pa (Tucker et al., 2014). The analytical characteristic shear stress of 0.027 Pa, which was calculated for the same rocking frequency and the same maximum tilt angle of 7°, was slightly lower compared to the time-averaged shear stress estimated with the finite element model.

*In vitro*, fluid flow generated by “see-saw” rockers has been shown to increase ALP activity and deposition of mineralized matrix in MSCs (Delaine-Smith et al., 2012) and to increase collagen secretion in tenocytes (Tucker et al., 2014). In another experiment, conditioned media from “rocked” osteocytes has upregulated osteogenic genes in MSCs (Hoey et al., 2011).

Oscillatory flow profiles can also be generated by orbital shakers which are described in more detail in Salek et al. (2012). Orbital shakers have been applied in bone mechanotransduction research, for example, when investigating osteoblast attachment (Aryaei and Jayasuriya, 2015), focal adhesion kinease functions (Young et al., 2011), and osteoblastic differentiation of mesenchymal stem cells (Lim et al., 2014).

#### 2.3.3 3D Perfusion Bioreactors

Different scaffold materials have been used for 3D bone cell culture aiming to provide a more natural cell environment compared to flat plastic or glass surfaces. Scaffolds have been manufactured using a wide range of different materials such as metals, ceramics, synthetic and natural polymers, and composites which are combinations of these materials. Scaffold composition and architecture intend to mimic the natural properties of bone including its porosity and stiffness. These properties are not only determined by the scaffold material itself but also by its fabrication process. Some of the most common methods to generate porous scaffolds are solvent casting, gas foaming, phase separation, freeze-drying, rapid-prototyping, and sintering (Karageorgiou and Kaplan, 2005; Stevens et al., 2008). Examples of scaffolds for bone cell applications include titanium fiber meshes (Bancroft et al., 2002), chitosan scaffolds (Su et al., 2014), polymerized high internal phase emulsions (PolyHIPEs) (Owen et al., 2016), and non-fibrillar hydrogels, such as poly(ethylene glycol) (PEG) (Chatterjee et al., 2010). Furthermore, collagen, which is a natural hydrogel, has been applied extensively in models mimicking the fibrillar, collageneous osteoid matrix (Parreno et al., 2008; Prideaux et al., 2014). Properties of bone scaffold materials have been reviewed in more detail for instance by Hutmacher (2000), Stevens et al. (2008), and Bose et al. (2012).

Several bioreactor types have been developed to combine fluid flow with 3D culture, including spinner flasks, rotating wall bioreactors, and perfusion systems (McCoy and O’Brien, 2010). The latter being most advantageous because a homogeneous microenvironment is generated within the porous scaffold by forcing fluid through the entire construct (Bancroft et al., 2002). Shear stress magnitudes within the scaffold can be approximated using the cylindrical pore model which takes into consideration the geometry of the scaffold and its porosity. The mean velocity through the pores *V_m_* can be calculated using the following equation:

(3) $$V_{m}=\frac{Q}{\phi \pi {\left(D/2\right)}^{2}}$$

where *Q* is the perfusion rate, *ø* is the porosity, and *D* is the diameter of the scaffold. Assuming a parabolic flow profile and cylindrical pores, the resulting shear stress at the scaffold wall *τ*, can be calculated:

(4) $$\tau =\frac{8{\mathrm{\mu V}}_{m}}{d}$$

where *μ* is the media viscosity and *d* is the diameter of the cylindrical pores in the scaffold (Goldstein, 2001).

In scaffolds, FSS is most likely not only caused by direct flow perfusion but it might also be an indirect product of scaffold strain (Sumanasinghe et al., 2006) and compression (Sittichockechaiwut et al., 2009; Vazquez et al., 2014). This behavior has been observed in natural bone where FSS is primarily a result of bone matrix deformation (Weinbaum et al., 1994). However, estimating FSS resulting from matrix strain and compression is not straight-forward. Moreover, a direct comparison of estimated shear stress magnitudes between monolayer and 3D experiments is often not relevant as the mechanical environment in 3D is more complex. For example, shear stress in 3D can also be transmitted to ECM fibers leading to ECM strain and thus cytoskeletal strain transmitted via integrins which can further increase the stress sensed by cells in 3D (Ng and Swartz, 2003).

### 2.4 Bone Cell Models for *In Vitro* Mechanotransduction Research

Advancement in bone mechanotransduction research depends on the availability of suitable cell model systems which can sense and respond to mechanical stimuli similar to cells *in vivo*. Bone cells can be sourced from different origins including

- pluripotent stem cells which are differentiating to osteoblasts (Jaiswal et al., 1997),
- primary osteoblasts and osteocytes from different species (Jonsson et al., 2009; Prideaux et al., 2016), and
- immortalized and osteosarcoma cell lines (Kato et al., 2001; Woo et al., 2011).

Each cell model has its own advantages and disadvantages; hence, the appropriate cell model has to be selected depending on the aim of the research (Czekanska et al., 2012).

#### 2.4.1 Osteoblast Cell Models

Primary osteoblasts have been successfully isolated and cultured from human bones (Jonsson et al., 2009). Human-derived cells are a good candidate for clinical research since outcomes are not influenced by interspecies differences. However, primary human osteoblasts are a heterogeneous population and their behavior depends on several factors, including isolation method (Voegele et al., 2000), cell location within the skeleton, and donor age (Martínez et al., 1999). All of these factors can be better controlled by isolating primary cells from animals, including rats (Orriss et al., 2012) and mice (Bakker and Klein-Nulend, 2012). In addition, animal-derived primary cells are more easily accessible and available in greater quantities (Czekanska et al., 2012).

Compared to primary cells, cell lines can provide even more homogeneous cell populations. They are either generated from immortalized primary cells, e.g., MC3T3-E1, human osteoblast-like cells (hOB), and human fetal osteoblast-like cells (hFOB), or they are derived from osteosarcomas, e.g., MG-63 and SaOS2 (Kartsogiannis and Ng, 2004).

A commonly used cell line in bone mechanotransduction research is MC3T3-E1 (Owan et al., 1997; You et al., 2001a; Genetos et al., 2004). This cell line was originally derived from primary cells collected from newborn mouse calvaria. MC3T3-E1 represents a preosteoblastic cell type capable of differentiating into osteoblasts, and deposits collagen and mineral nodules *in vitro* (Sudo et al., 1983; Franceschi and Iyer, 1992). However, variations in culture condition might have resulted in the formation of multiple sublines of this cell line. Studies comparing cells obtained from different sources found great differences in cell behavior, for example, regarding mineralization (Wang et al., 1999) and PGE_2_ production (Leis et al., 1997). These alterations in cell behavior should be considered when comparing results of different studies which are potentially not using the same MC3T3-E1 subclones but subclones in different differentiation stages.

The MLO-A5 cell line also originates from primary mice cells and has been used primarily in long-term studies investigating the mineralized matrix deposition (Delaine-Smith et al., 2012). In contrast to MC3T3-E1, MLO-A5 represent a post-osteoblast/preosteocyte cell type which more rapidly mineralizes (Kato et al., 2001). MLO-A5 express typical late-osteoblast markers, such as high ALP, bone sialoprotein (BSP), and osteocalcin (OCN) (Stern et al., 2012).

Osteosarcoma cells have also been used as a cell model when researching bone mechanotransduction (Myers et al., 2007). The cell line MG-63, for example, was originally derived from an osteogenic sarcoma of a 14-year-old male (Heremans et al., 1978). Osteosarcoma cell lines can be a valuable tool for investigating specific aspects of bone cell function such as cell adhesion. However, in certain aspects they behave very different to normal bone cells, specifically their growth characteristics and ALP activities differ considerably from primary osteoblasts (Clover and Gowen, 1994).

Moreover, mesenchymal stem cells (MSCs) can also be used as osteoblast cell models. Since MSCs are preosteoblasts, they are a good model for gaining a better understanding of the mechanisms guiding MSC differentiation. To induce osteogenic behavior in MSCs *in vitro*, they are commonly cultured in dexamethasone-supplemented culture medium (Kreke and Goldstein, 2004; Scaglione et al., 2008).

#### 2.4.2 Osteocyte Cell Models

Although osteocytes are the most abundant cell type in bone, their isolation and culture is very challenging due to their location deep within the bone matrix. Primary osteocytes were first successfully isolated from chicken calvariae. The cell isolation process involved digestion of chick bones with collagenase and EDTA, followed by purification of the heterogeneous cell population with an osteocyte specific antibody (van der Plas and Nijweide, 1992). Similar procedures were applied for the isolation of primary osteocytes from mice (Stern et al., 2012), rats (Gu et al., 2006), and most recently humans (Prideaux et al., 2016). However, primary osteocyte culture still faces many obstacles. For example, the yield of osteocytes after isolation is normally low and since they are terminally differentiated cells they also lack the capability to proliferate in culture (Stern et al., 2012).

The first osteocyte cell line was developed by Kato et al. (1997). They isolated the cell line MLO-Y4 from the long bones of transgenic mice. This cell line can be considered as the most common osteocyte cell model and it has been widely applied in mechanotransduction studies (Zhang et al., 2006; Ponik et al., 2007; Li et al., 2012). MLO-Y4 cells represent an early osteocyte cell type. Unlike osteoblasts, MLO-Y4 cells produce large amounts of osteocalcin but low levels of ALP and collagen. In addition, MLO-Y4 cells are characterized by extensive dendrites and increased expression of connexin 43 as expected in osteocytes (Kato et al., 1997). In contrast, MLO-Y4 do not express the genes dentin matrix protein 1 (DMP-1) and Sclerostin (Sost), which are normally found in osteocytes (Stern et al., 2012).

This short-coming has been overcome by the development of the osteoblast-to-late-osteocyte cell line IDG-SW3. In the beginning of culture, IDG-SW3 show characteristics of osteoblasts, e.g., expression of ALP and deposition of collagen type I and mineral. Over time, IDG-SW3 cells differentiate toward early osteocytes (expression of E11/gp38, Dmp1, Phex) and subsequently toward late osteocytes which possess a characteristic dendritic morphology and express SOST/sclerostin and FGF23 (Woo et al., 2011). IDG-SW3 were isolated from long bones of transgenic mice in which the Dmp1 promoter drives the expression of the green fluorescent protein (GFP), which allows observation of osteocytogenesis by fluorescence microscopy. The more recently developed osteocyte cell line Ocy454 was isolated from the same transgenic mouse model as IDG-SW3. In contrast to IDG-SW3, Ocy454 demonstrated osteocytic characteristics within a considerably shorter period of time and did not require specific differentiation medium containing ascorbic acid or *β*-glycerophosphate (Spatz et al., 2015).

### 2.5 Responses of Osteoblasts to Fluid Shear Stress

Osteoblasts respond to a wide range of different shear stimuli *in vitro* (Reich and Frangos, 1991; Owan et al., 1997; Bakker et al., 2001; Yu et al., 2014).

#### 2.5.1 Responses to High Fluid Shear Stress

Relatively high FSS ranging between 0.5 and 2 Pa have been widely reported to impact osteoblasts *in vitro*, including changes in biochemical factors and gene expression (Table 1). For example, flow shear rapidly increases intracellular calcium (Hung et al., 1995; You et al., 2001a), inositol trisphosphate (Reich and Frangos, 1991), nitric oxide (NO) (Johnson et al., 1996; Owan et al., 1997), prostaglandin E_2_ (PGE_2_) (Reich and Frangos, 1991; Klein-Nulend et al., 1997; Bakker et al., 2001), and adenosine triphosphate (ATP) (Genetos et al., 2004) levels in osteoblast cultures. PGE_2_, NO, and ATP have all been shown to depend on calcium signaling. Fluid flow has also been shown to regulate expression of osteoblast genes for osteopontin (OPN) (Owan et al., 1997; You et al., 2001a), cyclooxygenase-2 (COX-2), and c-FOS (Pavalko et al., 1998) but also genes related to matrix metabolism such as collagen I (Myers et al., 2007).

**Table 1:** **Osteoblastic responses to medium and high fluid shear stress**.

Cell type	Shear stress (Pa)	Flow type	Flow time	Culture system	mRNA	Other factors	Reference
Rat	0.6	s	12 h	PPFC		NO ↑	Johnson et al. (1996)
Rat	0.1–2.4	s, p	30 min	PPFC		*PGE*_2_ ↑	Reich and Frangos (1991)
Mouse	0.7	p	1 h	PPFC		*PGE*_2_ ↑	Klein-Nulend et al. (1997)
MC3T3	–	–	72 h	Bending device	OPN ↑		Owan et al. (1997)
Mouse	0.4–1.2	p	15 min	PPFC		NO ↑, *PGE*_2_ ↑	Bakker et al. (2001)
MC3T3	2	o	2 h	PPFC	OPN ↑	*Ca*^2+^ ↑	You et al. (2001a)
MC3T3	1.2	s	1 h	PPFC	COX-2 ↑, c-FOS ↑		Pavalko et al. (1998)
MC3T3	1.2	s	5 min	PPFC		*PGE*_2_ ↑, ATP ↑	Genetos et al. (2004)
MG-63	1	p	12 h	PPFC^a^	Col I ↑		Myers et al. (2007)

*Cell types refer to primary cells unless a specific cell line is stated. In case of oscillatory or pulsatile fluid flow the value given refers to the peak shear stress*.

*^a^A Streamer™ unit was used*.

*s, steady; p, pulsatile; o, oscillatory*.

Most experiments have been performed in PPFC, but the short flow durations indicate the previously discussed challenges of culturing cells in channels for more than a couple of hours (Zheng et al., 2010). In addition, osteoblast proliferation and matrix deposition will change the channel geometry over time and thus alter the shear stress (Nauman et al., 2001).

#### 2.5.2 Responses to Low Fluid Shear Stress

As discussed previously, osteoblasts unlike osteocytes might be more commonly subjected to low FSS (Figure 5C). Osteoblast responses to low FSS below 0.5 Pa have also been shown *in vitro* (Liegibel et al., 2004; Yu et al., 2014; Aisha et al., 2015). Responses of osteoblasts, which were cultured on planar surfaces as monolayer, to low FSS were comparable to high FSS responses (Table 2). It should be noted, however, that fewer studies investigated the lower stress range. Low FSS resulted in an increased production of PGE_2_ (Liegibel et al., 2004), ALP (Xing et al., 2014; Aisha et al., 2015), and collagen (Xing et al., 2014). Furthermore, osteoblasts tended to proliferate more under low flow (Liegibel et al., 2004; Xing et al., 2014; Aisha et al., 2015). mRNA expression of osteogenic genes Runx2, ALP, Col I, and osteocalcin was also increased under low FSS (Yu et al., 2014). Similar to high FSS experiments, PPFC were often used to provide the required stimuli in low FSS applications (Kou et al., 2011; Xing et al., 2014). In addition, rockers and orbital shakers were also used to generate FSS in this range (Liegibel et al., 2004; Aisha et al., 2015).

**Table 2:** **Osteoblastic responses to low fluid shear stress**.

Cell type	Shear stress	Flow type	Flow time	Culture system	mRNA	Other responses	Reference
hOB	1–63 *μ*Pa	o	10–96 h	Orbital shaker	ALP ↑	FN ↑, PGE_2_ ↑, TGF-*β* ↑, CP ↑	Liegibel et al. (2004)
Rat	0.03–0.3 Pa	s	2 min	PPFC		Ca^2+^ ↑	Kou et al. (2011)
Rat	0.5 Pa	s	24 h	PPFC	Col I ↑	FN ↑, ALP ↓, coll. ↑, CP ↑	Xing et al. (2014)
MC3T3	1.5–412 *μ*Pa	s	24 h	PPFC	ALP ↑, OCN ↑, Col I ↑, Runx2 ↑, OSX ↔	CP ↑	Yu et al. (2014)
NHOst	–	o	72 h	Orbital shaker		CP ↑, ALP ↑, OCN ↔	Aisha et al. (2015)

*Cell types refer to primary cells unless a specific cell line is stated. In case of oscillatory fluid flow, the value given refers to the peak shear stress*.

*s, steady; o, oscillatory; CP, cell proliferation; OSX, osterix; NHOst, normal human osteoblast*.

#### 2.5.3 Long-Term Responses by Cell Monolayers

Osteoblasts respond within hours to fluid flow by releasing signaling factors, including NO and prostaglandins (Bakker et al., 2001). However, it takes days to weeks for osteoblasts to begin laying down a collagen matrix which later mineralizes. For example, MLO-A5 or MC3T3-E1 require at least 3 days (Kato et al., 2001) or even 2 weeks (Fratzl-Zelman et al., 1998), respectively, before they begin to mineralize. Evaluation of such late responses – in addition to early responses – is important for a better understanding of bone remodeling and improved tissue engineering strategies. Bone strength, for example, is directly influenced by long-term responses, such as mineral content and collagen fiber distribution, but only indirectly linked to signaling factors, such as NO (Bouxsein and Seeman, 2009).

Due to the previously discussed difficulties in culturing cell for days and weeks under controlled flow conditions, only a small number of studies are looking into downstream responses of osteoblasts (Table 3). Morris et al. (2010) cultured MLO-A5 osteoblasts in commercially available PPFC for 10 days while applying fairly high intermittent unidirectional fluid flow (0.8 Pa). They did not report any of the previously reported technical difficulties associated with long-term culture in PPFC. Similarly, Scaglione et al. (2008) were able to culture cells continuously over 10 days in a PPFC, but their system consisted of higher channels (2 vs 0.4 mm), which facilitated long-term cell culture without obstructing the channels. However, high channels meant that only low FSS in the millipascal range could be generated with this system. Other studies apply flow only for a couple of minutes to a maximum of several hours before disassembling the chambers and continuing cell culture outside of the flow chambers (Kreke et al., 2005, 2008). Less defined and very low flow was also successfully applied by Delaine-Smith et al. (2012), who generated fluid flow in six-well plates with a see-saw rocker over 21 days.

**Table 3:** **Long-term effects or fluid flow on osteoblasts**.

Cell type	Shear stress	Flow type	Culture time (days)	Culture system	mRNA	Other responses	Reference
Rat MSC	0.06–0.6 Pa	p	24	PPFC		MZ ↔, PGE_2_ ↑	Nauman et al. (2001)
Rat MSC	0.036–0.27 Pa	s	21	RFC		CP ↔, OCN ↑, PGE_2_ ↑, no MZ	Kreke and Goldstein (2004)
Rat MSC	0.16 Pa	s	20	PPFC	OPN ↑, BSP ↑	CP ↔, ALP ←, OCN ↑	Kreke et al. (2005)
Rat MSC	0.23 Pa	s	14	PPFC	OCN ↑, OPN ↑, Col I ↑, COX-2 ↑, BSP ↑		Kreke et al. (2008)
Human MSC	1.2 mPa	s	10	PPFC	ALP ↓, OCN ↓, OPN ↔, COL I ↑, OSX ↑, cbfa-1 ↑, BSP ↔	CP ↓, coll. ↑, MZ ↑	Scaglione et al. (2008)
MLO-A5	0.051 Pa	o	21	Rocker		CP ↔, coll. ↑, MZ ↑, ALP ↔	Delaine-Smith et al. (2012)
MC3T3	1.2 Pa	s	12	PPFC	ALP ↑, Runx2 ↑, COX-2 ↑, BMP-2 ↑	coll. ↑, MZ ↑, ALP ↑	Mai et al. (2013a,b)
MLO-A5	0.051 Pa	o	12	Rocker		CP ↔, MZ ↑	Delaine-Smith et al. (2014)

*Cell types refer to primary cells unless a specific cell line is stated. In case of oscillatory or pulsatile fluid flow the value given refers to the peak shear stress*.

*s, steady; o, oscillatory; p, pulsatile; CP, cell proliferation; MZ, mineralization; coll., collagen; OSX, osterix*.

The few long-term monolayer studies available report conflicting results whether fluid shear stress can indeed increase collagen deposition and mineralization. Nauman et al. (2001) reported no change in mineralization under pulsatile fluid flow. Other studies show a significant increase in collagen and calcium deposition as a result of fluid flow (Scaglione et al., 2008; Morris et al., 2010; Delaine-Smith et al., 2012). Scaglione et al. (2008) reported, in the same study, a lower mRNA expression of OCN under fluid stimulation. OCN is a late phenotypic marker of osteoblastic differentiation and associated with mineralization. In contrast, Kreke and Goldstein (2004) report higher levels of OCN protein in the culture media when cells were exposed to high, intermittent shear stress. However, increased levels of OCN proteins did not translate directly to higher mineralization. Instead, the number of cell aggregates, where mineralization would initiate, decreased under fluid flow in the same study (Kreke and Goldstein, 2004).

#### 2.5.4 Long-Term Responses by Cells in 3D

In contrast to the few long-term flow studies available on flat surfaces, osteoblast behavior on scaffolds in 3D has been studied extensively (Table 4). *In vitro* experiments have demonstrated that perfusion increases osteogenic differentiation and production of calcified extracellular matrix in 3D (Bancroft et al., 2002; Sikavitsas et al., 2005). Perfusion bioreactor studies, however, were normally not able to distinguish between effects caused by an increased nutrient transport or the stimulatory effects of FSS on the cultured osteoblasts (Sikavitsas et al., 2005; Ban et al., 2011). It is possible to separate the effects of FSS and mass transport by changing the culture media’s viscosity (Sikavitsas et al., 2003; Li et al., 2009). For example, Sikavitsas et al. (2003) added dextran to the culture medium and demonstrated that enhanced osteogenic differentiation was indeed a result of increased FSS and not only greater mass transport.

**Table 4:** **Osteoblastic responses to fluid flow in 3D**.

Cell type	Shear stress	Culture time	Scaffold	mRNA	Other responses	Reference
Rat MSC	<0.1 Pa	16 days	Titanium fiber mesh		MZ ↑, OCN ↑, NO ↑	Bancroft et al. (2002)
Rat MSC	<0.03 Pa	16 days	Titanium fiber mesh		ALP ↑	Sikavitsas et al. (2003)
MC3T3	n.i.	7 days	Bone	ALP ↑, OCN ↑, Runx2 ↑	CP ↑	Cartmell et al. (2003)
Rat MSC	0.005 Pa	16 days	PLLA		MZ ↑, CP↑	Sikavitsas et al. (2005)
MC3T3	0.3 Pa	4 h	Porous collagen	Col I ↑		Tanaka et al. (2005)
Rat MSC	n.i.	16 days	Bone and titanium fibers		MZ ↑	Datta et al. (2006)
MC3T3	0.07–0.6 Pa	13 days	PDMS		ALP ↑, CP ↓	Leclerc et al. (2006)
MC3T3	n.i.	49 h	Collagen-GAG	OPN ↑, Col I ↑, COX-2 ↑	PGE_2_ ↑, CP ↓	Jaasma and O’Brien (2008)
Human MSC	0.005–0.015 Pa	28 days	*β*-TCP		MZ ↑, ALP ↑, OCN ↑	Li et al. (2009)
Rat	0.1 Pa	12 days	Bone	ALP ↑, OCN ↑	MZ ↑	Ban et al. (2011)
MG-63	n.i.	21 days	Chitosan	OCN ↑, Col I ↑	ALP ↑	Su et al. (2014)

*Cell types refer to primary cells unless a specific cell line is stated*.

*CP, cell proliferation; GAG, glycosaminoglycan; MZ, mineralization; n.i., not identified; PLLA, polylactic acid; TCP, tricalcium phosphate*.

FSS in perfusion bioreactors is commonly several magnitudes lower than shear stress generated with PPFC (Goldstein, 2001). However, exact estimation of FSS is difficult since shear stress profiles are often inhomogeneous depending on the porosity of the (irregular and often anisotropic) scaffolds. Furthermore, matrix deposition will alter the porosity and, therefore, change the magnitude of FSS over time.

### 2.6 Responses of Osteocytes to Fluid Shear Stress

Osteocytes comprise between 90 and 95% of bone cells and are embedded deeply within the mineralized bone matrix. Osteocytes were found to be extremely sensitive to mechanical loading and to respond by releasing soluble factors enabling them to control bone remodeling directly or via paracrine signaling (Bonewald, 2011).

#### 2.6.1 Direct Responses to Fluid Shear Stress

Osteocytes subjected to FSS have been shown to release several physiological relevant messengers *in vitro* (Table 5), including Ca^2+^ (Lu et al., 2012; Jing et al., 2013), ATP (Genetos et al., 2007), NO (Klein-Nulend et al., 1995a), and PGE_2_ (Klein-Nulend et al., 1997; Kamel et al., 2010).

**Table 5:** **Osteocytic responses to fluid flow**.

Cell type	Shear stress (Pa)	Flow type	Flow time	mRNA	Other responses	Reference
Chicken	0.5	p	45 min		NO ↑, PGE_2_ ↑	Klein-Nulend et al. (1995a)
Mouse	0.7	p	1 h		PGE_2_ ↑	Klein-Nulend et al. (1997)
Chicken	0.7	p	10 min		PGE_2_ ↑	Ajubi et al. (1999)
MLO-Y4^a^	0.4–1.62	s	2 h	E11 ↑		Zhang et al. (2006)
MLO-Y4	0.8, 1.1	s, o	24 h		OPN ↑, COX-2 ↑	Ponik et al. (2007)
MLO-Y4^a^	2	o	15 min		Ca^2+^ ↑, PGE_2_ ↑, ATP ↑	Genetos et al. (2007)
MLO-Y4	0.2–3.2	p	2 h		PGE_2_ ↑	Kamel et al. (2010)
MLO-Y4	0.5–5	o	4 h	COX-2 ↑, RANKL/OPG ↓		Li et al. (2012)
MLO-Y4	2	s, o			Ca^2+^ ↑	Lu et al. (2012)
MLO-Y4	1.6–3.2	s	24 h	Cx43 ↑, RANKL ↓, OPG ↑, Sost ↓	viability ↓	Li et al. (2013)
MLO-Y4^a^	0.5–4	s	10 min		Ca^2+^ ↑	Jing et al. (2013)
Ocy454^a^	0.05–0.8	s	2 h (3 days)	SOST ↓ RANKL ↓, 0.8 Pa: RANKL ↑ + Dmp1 ↑		Spatz et al. (2015)

*Cell types refer to primary cells unless a specific cell line is stated. In case of oscillatory or pulsatile fluid flow the value given refers to the peak shear stress*.

*s, steady; o, oscillatory; p, pulsatile*.

*^a^Studies which compare osteocytic responses to osteoblastic responses*.

FSS also regulates the release of RANKL and OPG. The relative abundance of RANKL compared to OPG is indicative of the amount of bone resorption. Downregulation of RANKL combined with upregulation of OPG as result of steady or oscillatory flow have been demonstrated in flow chamber experiments (Li et al., 2012, 2013). In addition, the mRNA expression of SOST/sclerostin, which functions as an inhibitor of bone formation, was reduced in short-term fluid flow studies (Spatz et al., 2015).

Osteocytes possess long dendritic processes which they use to form networks with neighboring osteocytes or cells on the bone surface (Kamioka et al., 2001). As a response to FSS, osteocytes are able to modify and enlarge this network. For example, Zhang et al. (2006) demonstrated that FSS increased dendricity and elongation of osteocyte dendrites because of a greater expression of E11 mRNA. The dendritic protein E11 is one of the earliest osteocyte markers appearing on the elongating dendritic processes. E11 is absent on primary osteoblasts and increases with time following differentiation into an osteocyte-like cell type.

Osteocytes communicate with each other and osteoblasts and osteoclasts on the bone surface primarily through gap junction channels also referred to as connexins. Connexin 43 (Cx43) is the most abundant gap junction channel on bone cells which can be found both on cell bodies and on their dendritic processes (Yellowley et al., 2000). Connexins allow small molecules, including Ca^2+^ and PGE_2_, to pass through and enable a coordinated response to external stimuli by osteocytes. Physiological FSS of 1.6 Pa enhanced osteogenesis through upregulation of Cx43, indicating the involvement of gap junctions in mechanical loading-induced signaling (Li et al., 2013).

#### 2.6.2 Paracrine Signaling between Osteocytes and Osteoblasts

Paracrine signaling enables osteocytes to control bone remodeling even though they cannot actively deposit or resorb bone matrix (Klein-Nulend et al., 2013). The release of signaling factors as a result of FSS (as summarized above) allows osteocytes to orchestrate osteoblast and osteoclast activity, which can be studied in coculture experiments (Table 6). Experimental set-ups include the culture of osteoblasts either (1) with conditioned media, i.e., culture media of previously mechanically stimulated osteocytes (Vezeridis et al., 2006; Hoey et al., 2011; Bakker et al., 2013) or (2) in transwell inserts allowing only indirect contact between cell types (Taylor et al., 2007).

**Table 6:** **Osteoblastic responses to paracrine signaling from osteocytes**.

Osteoblast	Osteocyte	Shear stress (Pa)	Flow type	Flow time (h)	Culture system	mRNA	Other responses	Reference
hFOB, MC3T3	MLO-Y4	0.44	–	1	Insert with rotating disk		ALP ↑	Taylor et al. (2007)
MC3T3, C3H10T1/2	MLO-Y4	0.05	o	2	Rocker	MC3T3: no changes; C3H10T1/2: COX-2 ↑, Runx2 ↔		Hoey et al. (2011)
Chicken	Chicken	0.7	p	1	PPFC		CP ↓, ALP ↑	Vezeridis et al. (2006)
MC3T3	MLO-Y4	0.7	p	1	PPFC	Runx2 ↓, OCN ↓, Ki67 ↑, DMP-1 ↔		Bakker et al. (2013)

*Cell types refer to primary cells unless a specific cell line is stated. In case of oscillatory or pulsatile fluid flow, the value given refers to the peak shear stress*.

*o, oscillatory; p, pulsatile; CP, cell proliferation*.

Coculture experiments demonstrated that osteocytes subjected to FSS regulate osteoblast proliferation (Vezeridis et al., 2006) and differentiation, for example, through the RANK ligand pathway (Hoey et al., 2011; Bakker et al., 2013). Osteocytes cultured both statically (Heino et al., 2004) and with fluid flow (Vezeridis et al., 2006; Taylor et al., 2007) induced enhanced ALP expression in osteoblasts.

In addition to the effect of FSS on paracrine signaling, Vazquez et al. (2014) demonstrated the impact of compression in a 3D coculture model. In their model, osteocytes were embedded in type I collagen gels with osteoblasts cultured on top of the hydrogel. Osteocytes formed networks within the 3D collagen gels and osteoblasts responded to mechanical loading with increased collagen production. The effects of dynamic compression were also investigated in a trabecular bone explant model by Chan et al. (2009). They observed increased PGE_2_ release by bone cells when osteoblasts were cocultured with osteocytes during a 4-week culture period.

## 3 Conclusion

**Figure 6:**
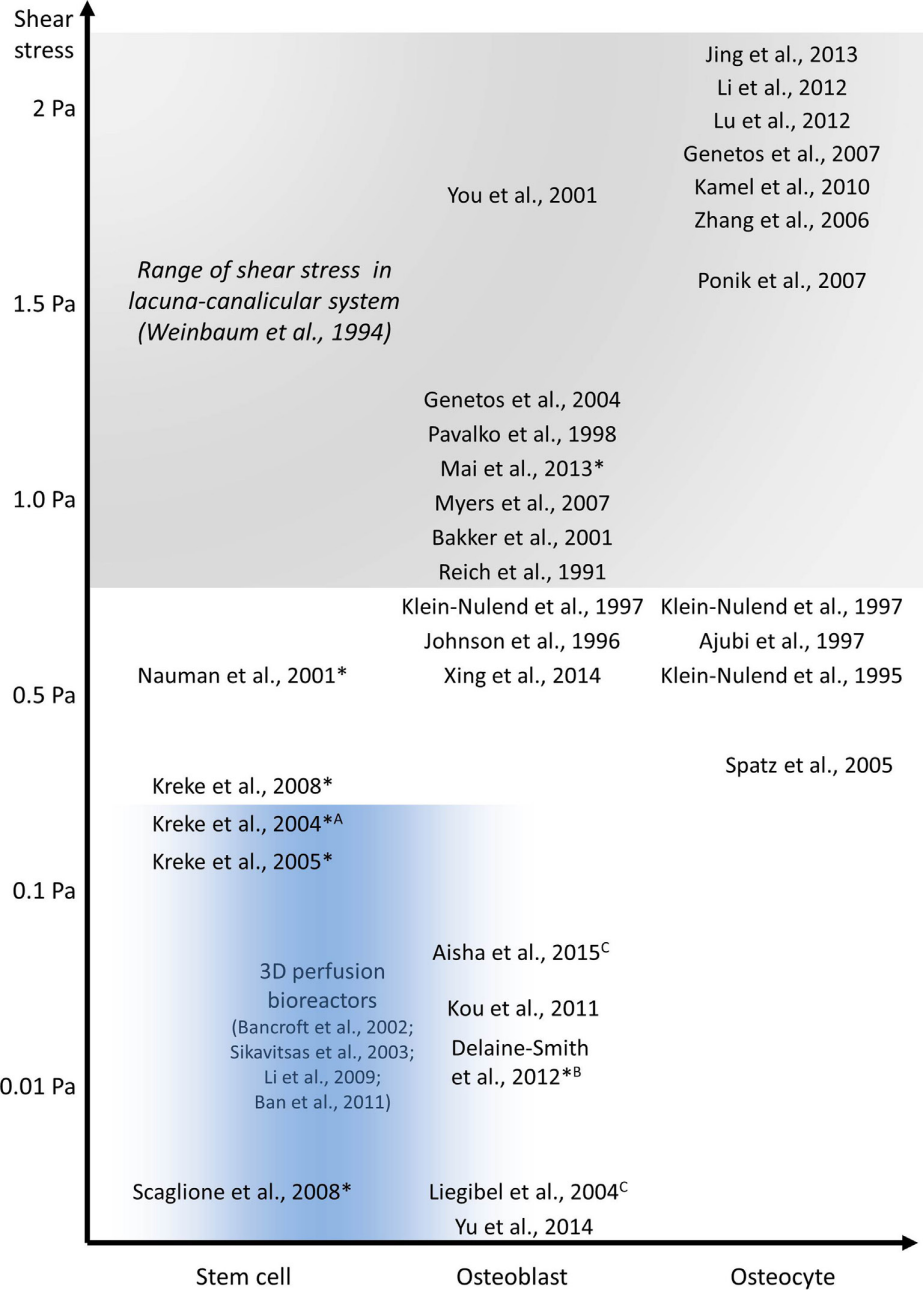
***In vitro* studies investigating the effect of fluid shear stress on osteoblast and osteocyte behavior**. All experiments were performed using PPFC, exceptions: ^A^radial flow chamber, ^B^rocking platform, ^C^orbital shaker, and 3D perfusion bioreactor experiments in blue area. Long-term experiments are indicated with *.

Osteoblasts and osteocytes respond to fluid shear stress over a wide range of magnitudes as demonstrated extensively *in vitro* (Figure 6). These responses indicate that fluid shear stress controls factors known to be important in bone formation. Different experimental set-ups and bone cell models have been applied over the last decades to investigate how fluid flow affects bone formation. These experiments have created a better understanding of how bone cells respond to different flow patterns and magnitudes of shear forces. For example, osteocytes respond to high fluid shear stress by releasing signaling factors which can also influence osteoblast behavior via paracrine signaling. The biomechanical environment of osteoblasts is less defined; hence, magnitudes of FSS ranging from only a few millipascals to more than 1 Pa have been investigated. Although early responses of bone cells are fairly well understood, more research is required into the long-term effects of FSS. Potentially, this requires the development of more sophisticated cell culture systems which can overcome the limitations of conventional parallel-plate flow chambers. A better understanding of collagen deposition and mineralization will aid the development of more reliable bone disease models and improved bone tissue engineering constructs which try to control the cell and collagen fiber orientation.

## Author Contributions

CW drafted the work. GR, DL, and CP revised and edited the manuscript. All the authors agree on the final version to be published.

## Conflict of Interest Statement

The authors declare that the research was conducted in the absence of any commercial or financial relationships that could be construed as a potential conflict of interest. The reviewer GV and handling Editor declared their shared affiliation, and the handling Editor states that the process nevertheless met the standards of a fair and objective review.

## Abbreviations

ALP, alkaline phosphatase; ATP, adenosine triphosphate; BMP-2, bone morphogenetic protein 2; BSP, bone sialoprotein; COX-2, cyclooxygenase-2; Cx43, connexin 43; DMP-1, dentin matrix protein 1; DNA, deoxyribonucleic acid; ECM, extracellular matrix; ER, endoplasmic reticulum; FGF-23, fibroblast growth factor 23; FSS, fluid shear stress; hFOB, human fetal osteoblast-like cells; hOB, human osteoblast-like cell; ISF, interstitial fluid; LCS, lacunar-canalicular system; M-CSF, macrophage colony-stimulating factor; MSC, mesenchymal stem cell; NO, nitric oxide; OCN, osteocalcin; OPG, osteoprotegerin; OPN, osteopontin; PIV, particle image velocimetry; PPFC, parallel-plate flow chamber; PGE_2_, prostaglandin E_2_; RANK, receptor activator of nuclear factor kappa-B; RANKL, receptor activator of nuclear factor kappa-B ligand; RNA, ribonucleic acid; Runx2, Runt-related transcription factor 2.

## References

[B1] Aisha M. D. Nor-Ashikin M. N. K. Sharaniza A. B. R. Nawawi H. Froemming G. R. A. (2015). Orbital fluid shear stress promotes osteoblast metabolism, proliferation and alkaline phosphates activity in vitro. Exp. Cell Res. 337, 87–93. 10.1016/j.yexcr.2015.07.002 26163894

[B2] Ajubi N. E. Klein-Nulend J. Alblas M. J. Burger E. H. Nijweide P. J. (1999). Signal transduction pathways involved in fluid flow-induced PGE2 production by cultured osteocytes. Am. J. Physiol. Endocrinol. Metab. 276, E171–E178. 9886964 10.1152/ajpendo.1999.276.1.E171

[B3] Al Nazer R. Lanovaz J. Kawalilak C. Johnston J. D. Kontulainen S. (2012). Direct in vivo strain measurements in human bone-a systematic literature review. J. Biomech. 45, 27–40. 10.1016/j.jbiomech.2011.08.004 21889149

[B4] Anderson E. J. Falls T. D. Sorkin A. M. Knothe Tate M. L. (2006). The imperative for controlled mechanical stresses in unraveling cellular mechanisms of mechanotransduction. Biomed. Eng. Online 5, 27. 10.1186/1475-925X-5-27 16672051 PMC1526737

[B5] Anderson H. C. (1995). Molecular biology of matrix vesicles. Clin. Orthop. Relat. Res. 314, 266–280. 7634645

[B6] Anderson H. C. (2003). Matrix vesicles and calcification. Curr. Rheumatol. Rep. 5, 222–226. 10.1007/s11926-003-0071-z 12744815

[B7] Aryaei A. Jayasuriya A. C. (2015). The effect of oscillatory mechanical stimulation on osteoblast attachment and proliferation. Mater. Sci. Eng. C 52, 129–134. 10.1016/j.msec.2015.03.024 25953549 PMC4443475

[B8] Bacabac R. G. Smit T. H. Cowin S. C. Van Loon J. J. W. A. Nieuwstadt F. T. M. Heethaar R. (2005). Dynamic shear stress in parallel-plate flow chambers. J. Biomech. 38, 159–167. 10.1016/j.jbiomech.2004.03.020 15519352

[B9] Bacabac R. G. Smit T. H. Mullender M. G. Dijcks S. J. Van Loon J. J. W. A. Klein-Nulend J. (2004). Nitric oxide production by bone cells is fluid shear stress rate dependent. Biochem. Biophys. Res. Commun. 315, 823–829. 10.1016/j.bbrc.2004.01.138 14985086

[B10] Bakker A. D. Klein-Nulend J. (2012). Osteoblast isolation from murine calvaria and long bones. Methods Mol. Biol. 816, 19–29. 10.1007/978-1-61779-415-5_2 22130919

[B11] Bakker A. D. Soejima K. Klein-Nulend J. Burger E. H. (2001). The production of nitric oxide and prostaglandin E2 by primary bone cells is shear stress dependent. J. Biomech. 34, 671–677. 10.1016/S0021-9290(00)00231-1 11311708

[B12] Bakker A. D. Zandieh-Doulabi B. Klein-Nulend J. (2013). Strontium ranelate affects signaling from mechanically-stimulated osteocytes towards osteoclasts and osteoblasts. Bone 53, 112–119. 10.1016/j.bone.2012.11.044 23234812

[B13] Ban Y. Wu Y.-Y. Yu T. Geng N. Wang Y.-Y. Liu X.-G. (2011). Response of osteoblasts to low fluid shear stress is time dependent. Tissue Cell 43, 311–317. 10.1016/j.tice.2011.06.003 21764096

[B14] Bancroft G. N. Sikavitsas V. I. van den Dolder J. Sheffield T. L. Ambrose C. G. Jansen J. A. (2002). Fluid flow increases mineralized matrix deposition in 3D perfusion culture of marrow stromal osteoblasts in a dose-dependent manner. Proc. Natl. Acad. Sci. U.S.A. 99, 12600–12605. 10.1073/pnas.202296599 12242339 PMC130506

[B15] Bijlani R. L. (2004). Understanding Medical Physiology, 3 Edn New Delhi: Jaypee Brothers Publishers.

[B16] Birk D. E. Nurminskaya M. V. Zycband E. I. (1995). Collagen fibrillogenesis in situ: fibril segments undergo post-depositional modifications resulting in linear and lateral growth during matrix development. Dev. Dyn. 202, 229–243. 10.1002/aja.1002020303 7780173

[B17] Bonewald L. F. (2011). The amazing osteocyte. J. Bone Miner. Res. 26, 229–238. 10.1002/jbmr.320 21254230 PMC3179345

[B18] Bonewald L. F. Johnson M. L. (2008). Osteocytes, mechanosensing and Wnt signaling. Bone 42, 606–615. 10.1016/j.bone.2007.12.224 18280232 PMC2349095

[B19] Bonivtch A. R. Bonewald L. F. Nicolella D. P. (2007). Tissue strain amplification at the osteocyte lacuna: a microstructural finite element analysis. J. Biomech. 40, 2199–2206. 10.1016/j.jbiomech.2006.10.040 17196968 PMC2094105

[B20] Bose S. Roy M. Bandyopadhyay A. (2012). Recent advances in bone tissue engineering scaffolds. Trends Biotechnol. 30, 546–554. 10.1016/j.tibtech.2012.07.005 22939815 PMC3448860

[B21] Boskey A. L. (2007). Mineralization of bones and teeth. Elements 3, 385–391. 10.2113/GSELEMENTS.3.6.385

[B22] Boskey A. L. (2013). Bone composition: relationship to bone fragility and antiosteoporotic drug effects. Bonekey Rep. 2, 447. 10.1038/bonekey.2013.181 24501681 PMC3909232

[B23] Bouxsein M. L. Seeman E. (2009). Quantifying the material and structural determinants of bone strength. Best Pract. Res. Clin. Rheumatol. 23, 741–753. 10.1016/j.berh.2009.09.008 19945686

[B24] Boyce B. F. Xing L. (2008). Functions of RANKL/RANK/OPG in bone modeling and remodeling. Arch. Biochem. Biophys. 473, 139–146. 10.1016/j.abb.2008.03.018 18395508 PMC2413418

[B25] Boyle W. J. Simonet W. S. Lacey D. L. (2003). Osteoclast differentiation and activation. Nature 423, 337–342. 10.1038/nature01658 12748652

[B26] Burger E. H. Klein-Nulend J. (1999). Mechanotransduction in bone – role of the lacuno-canalicular network. FASEB J. 13, 101–112. 10352151

[B27] Burra S. Nicolella D. P. Jiang J. X. (2011). Dark horse in osteocyte biology. Commun. Integr. Biol. 4, 48–50. 10.4161/cib.13646 21509177 PMC3073269

[B28] Canty E. G. Kadler K. E. (2005). Procollagen trafficking, processing and fibrillogenesis. J. Cell. Sci. 118(Pt 7), 1341–1353. 10.1242/jcs.01731 15788652

[B29] Caplan A. I. (1991). Mesenchymal stem cells. J. Orthop. Res. 9, 641–650. 10.1002/jor.1100090504 1870029

[B30] Caplan A. I. Bruder S. P. (2001). Mesenchymal stem cells: building blocks for molecular medicine in the 21st century. Trends Mol. Med. 7, 259–264. 10.1016/S1471-4914(01)02016-0 11378515

[B31] Cartmell S. H. Porter B. D. García A. J. Guldberg R. E. (2003). Effects of medium perfusion rate on cell-seeded three-dimensional bone constructs in vitro. Tissue Eng. 9, 1197–1203. 10.1089/10763270360728107 14670107

[B32] Chan M. E. Lu X. L. Huo B. Baik A. D. Chiang V. Guldberg R. E. (2009). A trabecular bone explant model of osteocyte-osteoblast co-culture for bone mechanobiology. Cell. Mol. Bioeng. 2, 405–415. 10.1007/s12195-009-0075-5 20827376 PMC2935082

[B33] Chatterjee K. Lin-Gibson S. Wallace W. E. Parekh S. H. Lee Y. J. Cicerone M. T. (2010). The effect of 3D hydrogel scaffold modulus on osteoblast differentiation and mineralization revealed by combinatorial screening. Biomaterials 31, 5051–5062. 10.1016/j.biomaterials.2010.03.024 20378163 PMC3125577

[B34] Clarke B. (2008). Normal bone anatomy and physiology. Clin. J. Am. Soc. Nephrol. 3(Suppl. 3), S131–S139. 10.2215/CJN.04151206 18988698 PMC3152283

[B35] Clover J. Gowen M. (1994). Are MG-63 and HOS TE85 human osteosarcoma cell lines representative models of the osteoblastic phenotype? Bone 15, 585–591. 10.1016/8756-3282(94)90305-0 7873286

[B36] Cowin S. C. Cardoso L. (2015). Blood and interstitial flow in the hierarchical pore space architecture of bone tissue. J. Biomech. 48, 842–854. 10.1016/j.jbiomech.2014.12.013 25666410 PMC4489573

[B37] Curtze S. Dembo M. Miron M. Jones D. B. (2004). Dynamic changes in traction forces with DC electric field in osteoblast-like cells. J. Cell. Sci. 117(Pt 13), 2721–2729. 10.1242/jcs.01119 15150319

[B38] Czekanska E. M. Stoddart M. J. Richards R. G. Hayes J. S. (2012). In search of an osteoblast cell model for in vitro research. Eur. Cell. Mater. 24, 1–17. 22777949 10.22203/ecm.v024a01

[B39] Dallas S. L. (2006). Dynamics of bone extracellular matrix assembly and mineralization. J. Musculoskelet. Neuronal. Interact. 6, 370–371. 17185829

[B40] Dallas S. L. Prideaux M. Bonewald L. F. (2013). The osteocyte: an endocrine cell. and more. Endocr. Rev. 34, 658–690. 10.1210/er.2012-1026 23612223 PMC3785641

[B41] Datta N. Pham Q. P. Sharma U. Sikavitsas V. I. Jansen J. A. Mikos A. G. (2006). In vitro generated extracellular matrix and fluid shear stress synergistically enhance 3D osteoblastic differentiation. Proc. Natl. Acad. Sci. U.S.A. 103, 2488–2493. 10.1073/pnas.0505661103 16477044 PMC1413766

[B42] Davies P. F. (1995). Flow-mediated endothelial mechanotransduction. Physiol. Rev. 75, 519–560. 7624393 10.1152/physrev.1995.75.3.519 PMC3053532

[B43] Delaine-Smith R. M. (2013). Mechanical and Physical Guidance of Osteogenic Differentiation and Matrix Production. Ph.D. thesis, University of Sheffield, Sheffield.

[B44] Delaine-Smith R. M. Javaheri B. Helen Edwards J. Vazquez M. Rumney R. M. H. (2015). Preclinical models for in vitro mechanical loading of bone-derived cells. Bonekey Rep. 4, 728. 10.1038/bonekey.2015.97 26331007 PMC4549923

[B45] Delaine-Smith R. M. MacNeil S. Reilly G. C. (2012). Matrix production and collagen structure are enhanced in two types of osteogenic progenitor cells by a simple fluid shear stress stimulus. Eur. Cell. Mater. 24, 162–174. 22865228 10.22203/ecm.v024a12

[B46] Delaine-Smith R. M. Sittichokechaiwut A. Reilly G. C. (2014). Primary cilia respond to fluid shear stress and mediate flow-induced calcium deposition in osteoblasts. FASEB J. 28, 430–439. 10.1096/fj.13-231894 24097311 PMC4012163

[B47] Ducy P. Zhang R. Geoffroy V. Ridall A. L. Karsenty G. (1997). Osf2/Cbfa1: a transcriptional activator of osteoblast differentiation. Cell 89, 747–754. 10.1016/S0092-8674(00)80257-3 9182762

[B48] Duncan R. L. Turner C. H. (1995). Mechanotransduction and the functional response of bone to mechanical strain. Calcif. Tissue Int. 57, 344–358. 10.1007/BF00302070 8564797

[B49] Feng Z. Wagatsuma Y. Kikuchi M. Kosawada T. Nakamura T. Sato D. (2014). The mechanisms of fibroblast-mediated compaction of collagen gels and the mechanical niche around individual fibroblasts. Biomaterials 35, 8078–8091. 10.1016/j.biomaterials.2014.05.072 24976242

[B50] Flynn B. P. Bhole A. P. Saeidi N. Liles M. Dimarzio C. A. Ruberti J. W. (2010). Mechanical strain stabilizes reconstituted collagen fibrils against enzymatic degradation by mammalian collagenase matrix metalloproteinase 8 (MMP-8). PLoS ONE 5:e12337. 10.1371/journal.pone.0012337 20808784 PMC2925882

[B51] Franceschi R. T. Iyer B. S. (1992). Relationship between collagen synthesis and expression of the osteoblast phenotype in MC3T3-E1 cells. J. Bone Miner. Res. 7, 235–246. 10.1002/jbmr.5650070216 1373931

[B52] Frangos J. Eskin S. McIntire L. Ives C. (1985). Flow effects on prostacyclin production by cultured human endothelial cells. Science 227, 1477–1479. 10.1126/science.3883488 3883488

[B53] Franz-Odendaal T. A. Hall B. K. Witten P. E. (2006). Buried alive: how osteoblasts become osteocytes. Dev. Dyn. 235, 176–190. 10.1002/dvdy.20603 16258960

[B54] Fratzl-Zelman N. Fratzl P. Hörandner H. Grabner B. Varga F. Ellinger A. (1998). Matrix mineralization in MC3T3-E1 cell cultures initiated by *β*-glycerophosphate pulse. Bone 23, 511–520. 10.1016/S8756-3282(98)00139-2 9855459

[B55] Freund J. B. Goetz J. G. Hill K. L. Vermot J. (2012). Fluid flows and forces in development: functions, features and biophysical principles. Development 139, 1229–1245. 10.1242/dev.073593 22395739 PMC3294433

[B56] Frost H. M. (1987). Bone “mass” and the “mechanostat”: a proposal. Anat. Rec. 219, 1–9. 10.1002/ar.1092190104 3688455

[B57] Frost H. M. (2003). Bone’s mechanostat: a 2003 update. Anat. Rec. A Discov. Mol. Cell. Evol. Biol. 275, 1081–1101. 10.1002/ar.a.10119 14613308

[B58] Garnero P. (2015). The role of collagen organization on the properties of bone. Calcif. Tissue Int. 97, 229–240. 10.1007/s00223-015-9996-2 25894071

[B59] Genetos D. C. Geist D. J. Liu D. Donahue H. J. Duncan R. L. (2004). Fluid shear-induced ATP secretion mediates prostaglandin release in MC3T3-E1 osteoblasts. J. Bone Miner. Res. 20, 41–49. 10.1359/JBMR.041009 15619668 PMC2929123

[B60] Genetos D. C. Kephart C. J. Zhang Y. Yellowley C. E. Donahue H. J. (2007). Oscillating fluid flow activation of gap junction hemichannels induces ATP release from MLO-Y4 osteocytes. J. Cell. Physiol. 212, 207–214. 10.1002/jcp.21021 17301958 PMC2929812

[B61] Gillespie P. G. Müller U. (2009). Mechanotransduction by hair cells: models, molecules, and mechanisms. Cell 139, 33–44. 10.1016/j.cell.2009.09.010 19804752 PMC2888516

[B62] Giraud-Guille M.-M. Belamie E. Mosser G. Helary C. Gobeaux F. Vigier S. (2008). Liquid crystalline properties of type I collagen: perspectives in tissue morphogenesis. Comptes Rendus Chimie 11, 245–252. 10.1016/j.crci.2007.05.005

[B63] Goldstein A. (2001). Effect of convection on osteoblastic cell growth and function in biodegradable polymer foam scaffolds. Biomaterials 22, 1279–1288. 10.1016/S0142-9612(00)00280-5 11336300

[B64] Golub E. E. (2009). Role of matrix vesicles in biomineralization. Biochim. Biophys. Acta 1790, 1592–1598. 10.1016/j.bbagen.2009.09.006 19786074 PMC2783689

[B65] Golub E. E. Boesze-Battaglia K. (2007). The role of alkaline phosphatase in mineralization. Curr. Opin. Orthop. 18, 444–448. 10.1016/0169-6009(92)90750-8

[B66] Gu G. Nars M. Hentunen T. A. Metsikkö K. Väänänen H. K. (2006). Isolated primary osteocytes express functional gap junctions in vitro. Cell Tissue Res. 323, 263–271. 10.1007/s00441-005-0066-3 16175387

[B67] Gundberg C. M. (2003). Matrix proteins. Osteoporos. Int. 14(Suppl. 5), S37–S40. 10.1007/s00198-003-1471-7 14504704

[B68] Hadjidakis D. J. Androulakis I. I. (2006). Bone remodeling. Ann. N. Y. Acad. Sci. 1092, 385–396. 10.1196/annals.1365.035 17308163

[B69] Han Y. Cowin S. C. Schaffler M. B. Weinbaum S. (2004). Mechanotransduction and strain amplification in osteocyte cell processes. Proc. Natl. Acad. Sci. U.S.A. 101, 16689–16694. 10.1073/pnas.0407429101 15539460 PMC534548

[B70] Harris A. K. Stopak D. Wild P. (1981). Fibroblast traction as a mechanism for collagen morphogenesis. Nature 290, 249–251. 10.1038/290249a0 7207616

[B71] Heck T. A. M. Wilson W. Foolen J. Cilingir A. C. Ito K. van Donkelaar C. C. (2015). A tissue adaptation model based on strain-dependent collagen degradation and contact-guided cell traction. J. Biomech. 48, 823–831. 10.1016/j.jbiomech.2014.12.023 25560271

[B72] Heino T. J. Hentunen T. A. Väänänen H. K. (2004). Conditioned medium from osteocytes stimulates the proliferation of bone marrow mesenchymal stem cells and their differentiation into osteoblasts. Exp. Cell Res. 294, 458–468. 10.1016/j.yexcr.2003.11.016 15023534

[B73] Heremans H. Billiau A. Cassiman J. J. Mulier J. C. de Somer P. (1978). In vitro cultivation of human tumor tissues. II. Morphological and virological characterization of three cell lines. Oncology 35, 246–252. 10.1159/000225298 218153

[B74] Hert J. Fiala P. Petrtýl M. (1994). Osteon orientation of the diaphysis of the long bones in man. Bone 15, 269–277. 10.1016/8756-3282(94)90288-7 8068447

[B75] Hillsley M. V. Frangos J. A. (1997). Alkaline phosphatase in osteoblasts is down-regulated by pulsatile fluid flow. Calcif. Tissue Int. 60, 48–53. 10.1007/s002239900185 9030480

[B76] Hoey D. A. Kelly D. J. Jacobs C. R. (2011). A role for the primary cilium in paracrine signaling between mechanically stimulated osteocytes and mesenchymal stem cells. Biochem. Biophys. Res. Commun. 412, 182–187. 10.1016/j.bbrc.2011.07.072 21810408 PMC3160132

[B77] Holmes D. F. Graham H. K. Kadler K. E. (1998). Collagen fibrils forming in developing tendon show an early and abrupt limitation in diameter at the growing tips. J. Mol. Biol. 283, 1049–1058. 10.1006/jmbi.1998.2153 9799643

[B78] Huesa C. Helfrich M. H. Aspden R. M. (2010). Parallel-plate fluid flow systems for bone cell stimulation. J. Biomech. 43, 1182–1189. 10.1016/j.jbiomech.2009.11.029 20031135

[B79] Hung C. T. Pollack S. R. Reilly T. M. Brighton C. T. (1995). Real-time calcium response of cultured bone cells to fluid flow. Clin. Orthop. Relat. Res. 313, 256–269. 7641488

[B80] Hutmacher D. W. (2000). Scaffolds in tissue engineering bone and cartilage. Biomaterials 21, 2529–2543. 10.1016/S0142-9612(00)00121-6 11071603

[B81] Ingber D. E. (2006). Cellular mechanotransduction: putting all the pieces together again. FASEB J. 20, 811–827. 10.1096/fj.05-5424rev 16675838

[B82] Irie K. Ejiri S. Sakakura Y. Shibui T. Yajima T. (2008). Matrix mineralization as a trigger for osteocyte maturation. J. Histochem. Cytochem. 56, 561–567. 10.1369/jhc.2008.950527 18319272 PMC2386767

[B83] Jaasma M. J. O’Brien F. J. (2008). Mechanical stimulation of osteoblasts using steady and dynamic fluid flow. Tissue Eng. Part A 14, 1213–1223. 10.1089/tea.2007.0321 18433309

[B84] Jacobs C. R. Yellowley C. E. Davis B. R. Zhou Z. Cimbala J. M. Donahue H. J. (1998). Differential effect of steady versus oscillating flow on bone cells. J. Biomech. 31, 969–976. 10.1016/S0021-9290(98)00114-6 9880053 PMC3057628

[B85] Jaiswal N. Haynesworth S. E. Caplan A. I. Bruder S. P. (1997). Osteogenic differentiation of purified, culture-expanded human mesenchymal stem cells in vitro. J. Cell. Biochem. 64, 295–312. 10.1002/(SICI)1097-4644(199702)64:2<295:AID-JCB12>3.0.CO;2-I 9027589

[B86] Jing D. Lu X. L. Luo E. Sajda P. Leong P. L. Guo X. E. (2013). Spatiotemporal properties of intracellular calcium signaling in osteocytic and osteoblastic cell networks under fluid flow. Bone 53, 531–540. 10.1016/j.bone.2013.01.008 23328496 PMC3594508

[B87] Johnson D. L. McAllister T. N. Frangos J. A. (1996). Fluid flow stimulates rapid and continuous release of nitric oxide in osteoblasts. Am. J. Physiol. Endocrinol. Metab. 271, E205–E208. 8760099 10.1152/ajpendo.1996.271.1.E205

[B88] Jones S. Boyde A. Pawley J. (1975). Osteoblasts and collagen orientation. Cell Tissue Res. 159, 73–80. 10.1007/BF00231996 1149092

[B89] Jonsson K. B. Frost A. Nilsson O. Ljunghall S. Ljunggren O. (2009). Three isolation techniques for primary culture of human osteoblast-like cells: a comparison. Acta Orthop. Scand. 70, 365–373. 10.3109/17453679908997826 10569267

[B90] Kadler K. E. Hill A. Canty-Laird E. G. (2008). Collagen fibrillogenesis: fibronectin, integrins, and minor collagens as organizers and nucleators. Curr. Opin. Cell Biol. 20, 495–501. 10.1016/j.ceb.2008.06.008 18640274 PMC2577133

[B91] Kagan H. M. Li W. (2003). Lysyl oxidase: properties, specificity, and biological roles inside and outside of the cell. J. Cell. Biochem. 88, 660–672. 10.1002/jcb.10413 12577300

[B92] Kamel M. A. Picconi J. L. Lara-Castillo N. Johnson M. L. (2010). Activation of β-catenin signaling in MLO-Y4 osteocytic cells versus 2T3 osteoblastic cells by fluid flow shear stress and PGE2: implications for the study of mechanosensation in bone. Bone 47, 872–881. 10.1016/j.bone.2010.08.007 20713195 PMC2952691

[B93] Kamioka H. Honjo T. Takano-Yamamoto T. (2001). A three-dimensional distribution of osteocyte processes revealed by the combination of confocal laser scanning microscopy and differential interference contrast microscopy. Bone 28, 145–149. 10.1016/S8756-3282(00)00421-X 11182371

[B94] Karageorgiou V. Kaplan D. (2005). Porosity of 3D biomaterial scaffolds and osteogenesis. Biomaterials 26, 5474–5491. 10.1016/j.biomaterials.2005.02.002 15860204

[B95] Kartsogiannis V. Ng K. W. (2004). Cell lines and primary cell cultures in the study of bone cell biology. Mol. Cell. Endocrinol. 228, 79–102. 10.1016/j.mce.2003.06.002 15541574

[B96] Kato Y. Boskey A. Spevak L. Dallas M. Hori M. Bonewald L. F. (2001). Establishment of an osteoid preosteocyte-like cell MLO-A5 that spontaneously mineralizes in culture. J. Bone Miner. Res. 16, 1622–1633. 10.1359/jbmr.2001.16.9.1622 11547831

[B97] Kato Y. Windle J. J. Koop B. A. Mundy G. R. Bonewald L. F. (1997). Establishment of an osteocyte-like cell line, MLO-Y4. J. Bone Miner. Res. 12, 2014–2023. 10.1359/jbmr.1997.12.12.2014 9421234

[B98] Katz E. P. Wachtel E. Yamauchi M. Mechanic G. L. (1989). The structure of mineralized collagen fibrils. Connect. Tissue Res. 21, 149–154; discussion 155–158. 10.3109/03008208909050005 2605938

[B99] Kerschnitzki M. Wagermaier W. Roschger P. Seto J. Shahar R. Duda G. N. (2011). The organization of the osteocyte network mirrors the extracellular matrix orientation in bone. J. Struct. Biol. 173, 303–311. 10.1016/j.jsb.2010.11.014 21081167

[B100] Khan A. F. Awais M. Khan A. S. Tabassum S. Chaudhry A. A. Rehman I. U. (2013). Raman spectroscopy of natural bone and synthetic apatites. Appl. Spectrosc. Rev. 48, 329–355. 10.1080/05704928.2012.721107

[B101] Kini U. Nandeesh B. N. (2012). “Physiology of bone formation, remodeling, and metabolism,” in Radionuclide and Hybrid Bone Imaging, eds Fogelman I. Gnanasegaran G. van der Wall H. (Berlin, Heidelberg: Springer), 29–57.

[B102] Klein-Nulend J. Bacabac R. G. Bakker A. D. (2012). Mechanical loading and how it affects bone cells: the role of the osteocyte cytoskeleton in maintaining our skeleton. Eur. Cell. Mater. 24, 278–291. 23007912 10.22203/ecm.v024a20

[B103] Klein-Nulend J. Bakker A. D. Bacabac R. G. Vatsa A. Weinbaum S. (2013). Mechanosensation and transduction in osteocytes. Bone 54, 182–190. 10.1016/j.bone.2012.10.013 23085083

[B104] Klein-Nulend J. Burger E. H. Semeins C. M. Raisz L. G. Pilbeam C. C. (1997). Pulsating fluid flow stimulates prostaglandin release and inducible prostaglandin G/H synthase mRNA expression in primary mouse bone cells. J. Bone Miner. Res. 12, 45–51. 10.1359/jbmr.1997.12.1.45 9240724

[B105] Klein-Nulend J. Semeins C. Ajubi N. Nijweide P. Burger E. (1995a). Pulsating fluid flow increases nitric oxide (NO) synthesis by osteocytes but not periosteal fibroblasts – correlation with prostaglandin upregulation. Biochem. Biophys. Res. Commun. 217, 640–648. 10.1006/bbrc.1995.2822 7503746

[B106] Klein-Nulend J. van der Plas A. Semeins C. M. Ajubi N. E. Frangos J. A. Nijweide P. J. (1995b). Sensitivity of osteocytes to biomechanical stress in vitro. FASEB J. 9, 441–445. 7896017 10.1096/fasebj.9.5.7896017

[B107] Knothe Tate M. L. Adamson J. R. Tami A. E. Bauer T. W. (2004). The osteocyte. Int. J. Biochem. Cell Biol. 36, 1–8. 10.1016/S1357-2725(03)00241-3 14592527

[B108] Knothe Tate M. L. Steck R. Forwood M. R. Niederer P. (2000). In vivo demonstration of load-induced fluid flow in the rat tibia and its potential implications for processes associated with functional adaptation. J. Exp. Biol. 203, 2737–2745. 10952874 10.1242/jeb.203.18.2737

[B109] Kou S. Pan L. van Noort D. Meng G. Wu X. Sun H. (2011). A multishear microfluidic device for quantitative analysis of calcium dynamics in osteoblasts. Biochem. Biophys. Res. Commun. 408, 350–355. 10.1016/j.bbrc.2011.04.044 21514277

[B110] Kreke M. R. Goldstein A. S. (2004). Hydrodynamic shear stimulates osteocalcin expression but not proliferation of bone marrow stromal cells. Tissue Eng. 10, 780–788. 10.1089/1076327041348455 15265295

[B111] Kreke M. R. Huckle W. R. Goldstein A. S. (2005). Fluid flow stimulates expression of osteopontin and bone sialoprotein by bone marrow stromal cells in a temporally dependent manner. Bone 36, 1047–1055. 10.1016/j.bone.2005.03.008 15869916

[B112] Kreke M. R. Sharp L. A. Lee Y. W. Goldstein A. S. (2008). Effect of intermittent shear stress on mechanotransductive signaling and osteoblastic differentiation of bone marrow stromal cells. Tissue Eng. Part A 14, 529–537. 10.1089/tea.2007.0068 18352827

[B113] Lamers E. Walboomers X. F. Domanski M. te Riet J. van Delft F. C. M. J. M. Luttge R. (2010). The influence of nanoscale grooved substrates on osteoblast behavior and extracellular matrix deposition. Biomaterials 31, 3307–3316. 10.1016/j.biomaterials.2010.01.034 20122723

[B114] Leblanc A. D. Schneider V. S. Evans H. J. Engelbretson D. A. Krebs J. M. (1990). Bone mineral loss and recovery after 17 weeks of bed rest. J. Bone Miner. Res. 5, 843–850. 10.1002/jbmr.5650050807 2239368

[B115] Leblond C. P. (1989). Synthesis and seretion of collagen by cells of connective tissue, bone and dentin. Anat. Rec. 224, 123–329. 10.1002/ar.1092240204 2672880

[B116] Leclerc E. David B. Griscom L. Lepioufle B. Fujii T. Layrolle P. (2006). Study of osteoblastic cells in a microfluidic environment. Biomaterials 27, 586–595. 10.1016/j.biomaterials.2005.06.002 16026825

[B117] Lee T. C. Taylor D. (1999). Bone remodelling: should we cry wolff? Ir. J. Med. Sci. 168, 102–105. 10.1007/BF02946474 10422387

[B118] Leis H. J. Hulla W. Gruber R. Huber E. Zach D. Gleispach H. (1997). Phenotypic heterogeneity of osteoblast-like MC3T3-E1 cells: changes of bradykinin-induced prostaglandin E2 production during osteoblast maturation. J. Bone Miner. Res. 12, 541–551. 10.1359/jbmr.1997.12.4.541 9101365

[B119] Li D. Tang T. Lu J. Dai K. (2009). Effects of flow shear stress and mass transport on the construction of a large-scale tissue-engineered bone in a perfusion bioreactor. Tissue Eng. Part A 15, 2773–2783. 10.1089/ten.TEA.2008.0540 19226211

[B120] Li J. Rose E. Frances D. Sun Y. You L. (2012). Effect of oscillating fluid flow stimulation on osteocyte mRNA expression. J. Biomech. 45, 247–251. 10.1016/j.jbiomech.2011.10.037 22119108

[B121] Li S. Van Den Diepstraten C. D’Souza S. J. Chan B. M. C. Pickering J. G. (2003). Vascular smooth muscle cells orchestrate the assembly of type I collagen via alpha2beta1 integrin, RhoA, and fibronectin polymerization. Am. J. Pathol. 163, 1045–1056. 10.1016/S0002-9440(10)63464-5 12937145 PMC1868248

[B122] Li X. Liu C. Li P. Li S. Zhao Z. Chen Y. (2013). Connexin 43 is a potential regulator in fluid shear stress-induced signal transduction in osteocytes. J. Orthop. Res. 31, 1959–1965. 10.1002/jor.22448 23878018

[B123] Liegibel U. M. Sommer U. Bundschuh B. Schweizer B. Hilscher U. Lieder A. (2004). Fluid shear of low magnitude increases growth and expression of TGFbeta1 and adhesion molecules in human bone cells in vitro. Exp. Clin. Endocrinol. Diabetes 112, 356–363. 10.1055/s-2004-821014 15239020

[B124] Lim K. T. Hexiu J. Kim J. Seonwoo H. Choung P.-H. Chung J. H. (2014). Synergistic effects of orbital shear stress on in vitro growth and osteogenic differentiation of human alveolar bone-derived mesenchymal stem cells. Biomed Res. Int. 2014, 1–18. 10.1155/2014/316803 PMC3914586 24575406

[B125] Lu X. L. Huo B. Park M. Guo X. E. (2012). Calcium response in osteocytic networks under steady and oscillatory fluid flow. Bone 51, 466–473. 10.1016/j.bone.2012.05.021 22750013 PMC3412915

[B126] Mai Z.-H. Peng Z.-L. Zhang J.-L. Chen L. Liang H.-Y. Cai B. (2013a). miRNA expression profile during fluid shear stress-induced osteogenic differentiation in MC3T3-E1 cells. Chin. Med. J. 126, 1544–1550. 10.3760/cma.j.issn.0366-6999.20123137 23595392

[B127] Mai Z. Peng Z. Wu S. Zhang J. Chen L. Liang H. (2013b). Single bout short duration fluid shear stress induces osteogenic differentiation of MC3T3-E1 cells via integrin β1 and BMP2 signaling cross-talk. PLoS ONE 8:e61600 10.1371/journal.pone.0061600 23593489 PMC3623893

[B128] Malone A. M. D. Anderson C. T. Tummala P. Kwon R. Y. Johnston T. R. Stearns T. (2007). Primary cilia mediate mechanosensing in bone cells by a calcium-independent mechanism. Proc. Natl. Acad. Sci. U.S.A. 104, 13325–13330. 10.1073/pnas.0700636104 17673554 PMC1939687

[B129] Manolagas S. C. (2000). Birth and death of bone cells: basic regulatory mechanisms and implications for the pathogenesis and treatment of osteoporosis. Endocr. Rev. 21, 115–137. 10.1210/edrv.21.2.0395 10782361

[B130] Martin R. Boardman D. (1993). The effects of collagen fiber orientation, porosity, density, and mineralization on bovine cortical bone bending properties. J. Biomech. 26, 1047–1054. 10.1016/S0021-9290(05)80004-1 8408087

[B131] Martin R. Lau S. Mathews P. Gibson V. Stover S. (1996). Collagen fiber organization is related to mechanical properties and remodeling in equine bone. A comparsion of two methods. J. Biomech. 29, 1515–1521. 10.1016/S0021-9290(96)80002-9 8945649

[B132] Martínez M. E. del Campo M. T. Medina S. Sánchez M. Sánchez-Cabezudo M. J. Esbrit P. (1999). Influence of skeletal site of origin and donor age on osteoblastic cell growth and differentiation. Calcif. Tissue Int. 64, 280–286. 10.1007/s002239900619 10089218

[B133] Matsugaki A. Aramoto G. Ninomiya T. Sawada H. Hata S. Nakano T. (2015a). Abnormal arrangement of a collagen/apatite extracellular matrix orthogonal to osteoblast alignment is constructed by a nanoscale periodic surface structure. Biomaterials 37, 134–143. 10.1016/j.biomaterials.2014.10.025 25453944

[B134] Matsugaki A. Isobe Y. Saku T. Nakano T. (2015b). Quantitative regulation of bone-mimetic, oriented collagen/apatite matrix structure depends on the degree of osteoblast alignment on oriented collagen substrates. J. Biomed. Mater. Res. A 103, 489–499. 10.1002/jbm.a.35189 24733774

[B135] Matsugaki A. Fujiwara N. Nakano T. (2013). Continuous cyclic stretch induces osteoblast alignment and formation of anisotropic collagen fiber matrix. Acta Biomater. 9, 7227–7235. 10.1016/j.actbio.2013.03.015 23523937

[B136] McCoy R. J. O’Brien F. J. (2010). Influence of shear stress in perfusion bioreactor cultures for the development of three-dimensional bone tissue constructs: a review. Tissue Eng. Part B Rev. 16, 587–601. 10.1089/ten.TEB.2010.0370 20799909

[B137] McDonald J. A. Kelley D. G. Broekelmann T. J. (1982). Role of fibronectin in collagen deposition: fab’ to the gelatin-binding domain of fibronectin inhibits both fibronectin and collagen organization in fibroblast extracellular matrix. J. Cell Biol. 92, 485–492. 10.1083/jcb.92.2.485 7061591 PMC2112086

[B138] McGarry J. G. Klein-Nulend J. Mullender M. G. Prendergast P. J. (2005). A comparison of strain and fluid shear stress in stimulating bone cell responses–a computational and experimental study. FASEB J. 19, 482–484. 10.1096/fj.04-2210fje 15625080

[B139] Morris H. L. Reed C. I. Haycock J. W. Reilly G. C. (2010). Mechanisms of fluid-flow-induced matrix production in bone tissue engineering. Proc. Inst. Mech. Eng. H. 224, 1509–1521. 10.1243/09544119JEIM751 21287834

[B140] Mullen C. A. Haugh M. G. Schaffler M. B. Majeska R. J. McNamara L. M. (2013). Osteocyte differentiation is regulated by extracellular matrix stiffness and intercellular separation. J. Mech. Behav. Biomed. Mater. 28, 183–194. 10.1016/j.jmbbm.2013.06.013 23994943 PMC5776008

[B141] Mullender M. El Haj A. J. Yang Y. van Duin M. A. Burger E. H. Klein-Nulend J. (2004). Mechanotransduction of bone cells in vitro: mechanobiology of bone tissue. Med. Biol. Eng. Comput. 42, 14–21. 10.1007/BF02351006 14977218

[B142] Myers K. A. Rattner J. B. Shrive N. G. Hart D. A. (2007). Osteoblast-like cells and fluid flow: cytoskeleton-dependent shear sensitivity. Biochem. Biophys. Res. Commun. 364, 214–219. 10.1016/j.bbrc.2007.09.109 17942076

[B143] Nair A. K. Gautieri A. Chang S.-W. Buehler M. J. (2013). Molecular mechanics of mineralized collagen fibrils in bone. Nat. Commun. 4, 1724. 10.1038/ncomms2720 23591891 PMC3644085

[B144] Nauman E. A. Satcher R. L. Keaveny T. M. Halloran B. P. Bikle D. D. (2001). Osteoblasts respond to pulsatile fluid flow with short-term increases in PGE2 but no change in mineralization. J. Appl. Physiol. 90, 1849–1854. 11299276 10.1152/jappl.2001.90.5.1849

[B145] Ng C. P. Hiny B. Swartz M. A. (2005). Interstitial fluid flow induces myofibroblast differentiation and collagen alignment in vitro. J. Cell. Sci. 118, 4731–4739. 10.1242/jcs.02605 16188933

[B146] Ng C. P. Swartz M. A. (2003). Fibroblast alignment under interstitial fluid flow using a novel 3-D tissue culture model. Am. J. Physiol. Heart Circ. Physiol. 284, H1771––H1777. 10.1152/ajpheart.01008.2002 12531726

[B147] Niu X. Wang L. Tian F. Wang L. Li P. Feng Q. (2016). Shear-mediated crystallization from amorphous calcium phosphate to bone apatite. J. Mech. Behav. Biomed. Mater. 54, 131–140. 10.1016/j.jmbbm.2015.09.024 26454356

[B148] Orimo H. (2010). The mechanism of mineralization and the role of alkaline phosphatase in health and disease. J. Nihon Med. Sch. 77, 4–12. 10.1272/jnms.77.4 20154452

[B149] Orriss I. R. Taylor S. E. B. Arnett T. R. (2012). Rat osteoblast cultures. Methods Mol. Biol. 816, 31–41. 10.1007/978-1-61779-415-5_3 22130920

[B150] Owan I. Burr D. B. Turner C. H. Qiu J. Tu Y. Onyia J. E. (1997). Mechanotransduction in bone: osteoblasts are more responsive to fluid forces than mechanical strain. Am. J. Physiol. Cell Physiol. 273, C810–C815. 9316399 10.1152/ajpcell.1997.273.3.C810

[B151] Owen R. Sherborne C. Paterson T. Green N. H. Reilly G. C. Claeyssens F. (2016). Emulsion templated scaffolds with tunable mechanical properties for bone tissue engineering. J. Mech. Behav. Biomed. Mater. 54, 159–172. 10.1016/j.jmbbm.2015.09.019 26458114 PMC4717122

[B152] Parreno J. Buckley-Herd G. De-Hemptinne I. Hart D. A. (2008). Osteoblastic MG-63 cell differentiation, contraction, and mRNA expression in stress-relaxed 3D collagen I gels. Mol. Cell. Biochem. 317, 21–32. 10.1007/s11010-008-9801-x 18566755

[B153] Pavalko F. M. Chen N. X. Turner C. H. Burr D. B. Atkinson S. Hsieh Y.-F. (1998). Fluid shear-induced mechanical signaling in MC3T3-E1 osteoblasts requires cytoskeleton-integrin interactions. Am. J. Physiol. Cell Physiol. 275, C1591–C1601. 9843721

[B154] Pedersen J. A. Lichter S. Swartz M. A. (2010). Cells in 3D matrices under interstitial flow: effects of extracellular matrix alignment on cell shear stress and drag forces. J. Biomech. 43, 900–905. 10.1016/j.jbiomech.2009.11.007 20006339

[B155] Poellmann M. J. Estrada J. B. Boudou T. Berent Z. T. Franck C. Wagoner Johnson A. J. (2015). Differences in morphology and traction generation of cell lines representing different stages of osteogenesis. J. Biomech. Eng. 137, 124503. 10.1115/1.4031848 26501398

[B156] Polishchuk R. S. Polishchuk E. V. Marra P. Alberti S. Buccione R. Luini A. (2000). Correlative light-electron microscopy reveals the tubular-saccular ultrastructure of carriers operating between Golgi apparatus and plasma membrane. J. Cell Biol. 148, 45–58. 10.1083/jcb.148.1.45 10629217 PMC2156208

[B157] Ponik S. M. Triplett J. W. Pavalko F. M. (2007). Osteoblasts and osteocytes respond differently to oscillatory and unidirectional fluid flow profiles. J. Cell. Biochem. 100, 794–807. 10.1002/jcb.21089 17031855

[B158] Prideaux M. Loveridge N. Pitsillides A. A. Farquharson C. (2012). Extracellular matrix mineralization promotes E11/gp38 glycoprotein expression and drives osteocytic differentiation. PLoS ONE 7:e36786. 10.1371/journal.pone.0036786 22586496 PMC3346717

[B159] Prideaux M. Schutz C. Wijenayaka A. R. Findlay D. M. Campbell D. G. Solomon L. B. (2016). Isolation of osteocytes from human trabecular bone. Bone 88, 64–72. 10.1016/j.bone.2016.04.017 27109824

[B160] Prideaux M. Wijenayaka A. R. Kumarasinghe D. D. Ormsby R. T. Evdokiou A. Findlay D. M. (2014). SaOS2 osteosarcoma cells as an in vitro model for studying the transition of human osteoblasts to osteocytes. Calcif. Tissue Int. 95, 183–193. 10.1007/s00223-014-9879-y 24916279

[B161] Prockop D. J. Sieron A. L. Li S. W. (1998). Procollagen N-proteinase and procollagen C-proteinase. Two unusual metalloproteinases that are essential for procollagen processing probably have important roles in development and cell signaling. Matrix Biol. 16, 399–408. 10.1016/S0945-053X(98)90013-0 9524360

[B162] Puustjärvi K. Nieminen J. Räsänen T. Hyttinen M. Helminen H. J. Kröger H. (1999). Do more highly organized collagen fibrils increase bone mechanical strength in loss of mineral density after one-year running training? J. Bone Miner. Res. 14, 321–329. 10.1359/jbmr.1999.14.3.321 10027896

[B163] Reich K. Frangos J. (1991). Effect of flow on prostaglandin E2 and inositol trisphosphate levels in osteoblasts. Am. J. Physiol. 261(3 Pt 1), C428–C432. 1887871 10.1152/ajpcell.1991.261.3.C428

[B164] Reilly G. C. Haut T. R. Yellowley C. E. Donahue H. J. Jacobs C. R. (2003). Fluid flow induced PGE2 release by bone cells is reduced by glycocalyx degradation whereas calcium signals are not. Biorheology 40, 591–603. 14610310

[B165] Riggs C. Vaughan L. Evans G. Lanyon L. Boyde A. (1993). Mechanical implications of collagen fibre orientation in cortical bone of the equine radius. Anat. Embryol. 187, 239–248. 10.1007/BF00195761 8470824

[B166] Rosenberg N. Rosenberg O. Soudry M. (2012). Osteoblasts in bone physiology-mini review. Rambam Maimonides Med. J. 3, e0013. 10.5041/RMMJ.10080 23908837 PMC3678809

[B167] Rubin J. Rubin C. Jacobs C. R. (2006). Molecular pathways mediating mechanical signaling in bone. Gene 367, 1–16. 10.1016/j.gene.2005.10.028 16361069 PMC3687520

[B168] Salek M. M. Sattari P. Martinuzzi R. J. (2012). Analysis of fluid flow and wall shear stress patterns inside partially filled agitated culture well plates. Ann. Biomed. Eng. 40, 707–728. 10.1007/s10439-011-0444-9 22042624

[B169] Scaglione S. Wendt D. Miggino S. Papadimitropoulos A. Fato M. Quarto R. (2008). Effects of fluid flow and calcium phosphate coating on human bone marrow stromal cells cultured in a defined 2D model system. J. Biomed. Mater. Res. A 86, 411–419. 10.1002/jbm.a.31607 17969030

[B170] Schulze E. Witt M. Kasper M. Löwik C. W. Funk R. H. (1999). Immunohistochemical investigations on the differentiation marker protein E11 in rat calvaria, calvaria cell culture and the osteoblastic cell line ROS 17/2.8. Histochem. Cell Biol. 111, 61–69. 10.1007/s004180050334 9930885

[B171] Schumacker P. T. (2002). Straining to understand mechanotransduction in the lung. Am. J. Physiol. Lung Cell Mol. Physiol. 282, L881–L882. 10.1152/ajplung.00043.2002 11943649

[B172] Scott J. E. (1995). Extracellular matrix, supramolecular organisation and shape. J. Anat. 187, 259–269. 7591990 PMC1167422

[B173] Seto J. Gupta H. S. Zaslansky P. Wagner H. D. Fratzl P. (2008). Tough lessons from bone: extreme mechanical anisotropy at the mesoscale. Adv. Funct. Mater. 18, 1905–1911. 10.1002/adfm.200800214

[B174] Shapiro F. (1988). Cortical bone repair. The relationship of the lacunar-canalicular system and intercellular gap junctions to the repair process. J. Bone Joint Surg. Am. 70, 1067–1081. 3042791

[B175] Shapiro F. (2008). Bone development and its relation to fracture repair. The role of mesenchymal osteoblasts and surface osteoblasts. Eur. Cell. Mater. 15, 53–76. 18382990 10.22203/ecm.v015a05

[B176] Sikavitsas V. I. Bancroft G. N. Holtorf H. L. Jansen J. A. Mikos A. G. (2003). Mineralized matrix deposition by marrow stromal osteoblasts in 3D perfusion culture increases with increasing fluid shear forces. Proc. Natl. Acad. Sci. U.S.A. 100, 14683–14688. 10.1073/pnas.2434367100 14657343 PMC299759

[B177] Sikavitsas V. I. Bancroft G. N. Lemoine J. J. Liebschner M. A. K. Dauner M. Mikos A. G. (2005). Flow perfusion enhances the calcified matrix deposition of marrow stromal cells in biodegradable nonwoven fiber mesh scaffolds. Ann. Biomed. Eng. 33, 63–70. 10.1007/s10439-005-8963-x 15709706

[B178] Sikavitsas V. I. Temenoff J. S. Mikos A. G. (2001). Biomaterials and bone mechanotransduction. Biomaterials 22, 2581–2593. 10.1016/S0142-9612(01)00002-3 11519777

[B179] Sittichockechaiwut A. Scutt A. M. Ryan A. J. Bonewald L. F. Reilly G. C. (2009). Use of rapidly mineralising osteoblasts and short periods of mechanical loading to accelerate matrix maturation in 3D scaffolds. Bone 44, 822–829. 10.1016/j.bone.2008.12.027 19442630

[B180] Spatz J. M. Wein M. N. Gooi J. H. Qu Y. Garr J. L. Liu S. (2015). The wnt inhibitor sclerostin is up-regulated by mechanical unloading in osteocytes in vitro. J. Biol. Chem. 290, 16744–16758. 10.1074/jbc.M114.628313 25953900 PMC4505423

[B181] Stern A. R. Stern M. M. Van Dyke M. E. Jähn K. Prideaux M. Bonewald L. F. (2012). Isolation and culture of primary osteocytes from the long bones of skeletally mature and aged mice. BioTechniques 52, 361–373. 10.2144/0000113876 22668415 PMC3612989

[B182] Stevens B. Yang Y. Mohandas A. Stucker B. Nguyen K. T. (2008). A review of materials, fabrication methods, and strategies used to enhance bone regeneration in engineered bone tissues. J. Biomed. Mater. Res. B 85B, 573–582. 10.1002/jbm.b.30962 17937408

[B183] Su W.-T. Wang Y.-T. Chou C.-M. (2014). Optimal fluid flow enhanced mineralization of MG-63 cells in porous chitosan scaffold. J. Taiwan Inst. Chem. Eng. 45, 1111–1118. 10.1016/j.jtice.2013.10.016

[B184] Suda T. Takahashi N. Udagawa N. Jimi E. Gillespie M. T. Martin T. J. (1999). Modulation of osteoclast differentiation and function by the new members of the tumor necrosis factor receptor and ligand families. Endocr. Rev. 20, 345–357. 10.1210/edrv.20.3.0367 10368775

[B185] Sudo H. Kodama H. A. Amagai Y. Yamamoto S. Kasai S. (1983). In vitro differentiation and calcification in a new clonal osteogenic cell line derived from newborn mouse calvaria. J. Cell Biol. 96, 191–198. 10.1083/jcb.96.1.191 6826647 PMC2112252

[B186] Sumanasinghe R. D. Bernacki S. H. Loboa E. G. (2006). Osteogenic differentiation of human mesenchymal stem cells in collagen matrices: effect of uniaxial cyclic tensile strain on bone morphogenetic protein (BMP-2) mRNA expression. Tissue Eng. 12, 3459–3465. 10.1089/ten.2006.12.3459 17518682

[B187] Swartz M. A. Fleury M. E. (2007). Interstitial flow and its effects in soft tissues. Annu. Rev. Biomed. Eng. 9, 229–256. 10.1146/annurev.bioeng.9.060906.151850 17459001

[B188] Tanaka S. M. Sun H. B. Roeder R. K. Burr D. B. Turner C. H. Yokota H. (2005). Osteoblast responses one hour after load-induced fluid flow in a three-dimensional porous matrix. Calcif. Tissue Int. 76, 261–271. 10.1007/s00223-004-0238-2 15812578

[B189] Taylor A. F. Saunders M. M. Shingle D. L. Cimbala J. M. Zhou Z. Donahue H. J. (2007). Mechanically stimulated osteocytes regulate osteoblastic activity via gap junctions. Am. J. Physiol. Cell Physiol. 292, C545–C552. 10.1152/ajpcell.00611.2005 16885390

[B190] Tomasek J. J. Gabbiani G. Hinz B. Chaponnier C. Brown R. A. (2002). Myofibroblasts and mechano-regulation of connective tissue remodelling. Nat. Rev. Mol. Cell Biol. 3, 349–363. 10.1038/nrm809 11988769

[B191] Tucker R. P. Henningsson P. Franklin S. L. Chen D. Ventikos Y. Bomphrey R. J. (2014). See-saw rocking: an in vitro model for mechanotransduction research. J. R. Soc. Interface 11, 20140330. 10.1098/rsif.2014.0330 24898022 PMC4208364

[B192] van der Plas A. Nijweide P. J. (1992). Isolation and purification of osteocytes. J. Bone Miner. Res. 7, 389–396. 10.1359/jbmr.2005.20.4.706 1609628

[B193] van der Rest M. Garrone R. (1991). Collagen family of proteins. FASEB J. 5, 2814–2823. 1916105

[B194] Vazquez M. Evans B. A. J. Riccardi D. Evans S. L. Ralphs J. R. Dillingham C. M. (2014). A new method to investigate how mechanical loading of osteocytes controls osteoblasts. Front. Endocrinol. 5:208. 10.3389/fendo.2014.00208 25538684 PMC4260042

[B195] Vezeridis P. S. Semeins C. M. Chen Q. Klein-Nulend J. (2006). Osteocytes subjected to pulsating fluid flow regulate osteoblast proliferation and differentiation. Biochem. Biophys. Res. Commun. 348, 1082–1088. 10.1016/j.bbrc.2006.07.146 16904067

[B196] Vico L. Collet P. Guignandon A. Lafage-Proust M.-H. Thomas T. Rehailia M. (2000). Effects of long-term microgravity exposure on cancellous and cortical weight-bearing bones of cosmonauts. Lancet 355, 1607–1611. 10.1016/S0140-6736(00)02217-0 10821365

[B197] Viguet-Carrin S. Garnero P. Delmas P. D. (2006). The role of collagen in bone strength. Osteoporos. Int. 17, 319–336. 10.1007/s00198-005-2035-9 16341622

[B198] Voegele T. J. Voegele-Kadletz M. Esposito V. Macfelda K. Oberndorfer U. Vecsei V. (2000). The effect of different isolation techniques on human osteoblast-like cell growth. Anticancer Res. 20, 3575–3581. 11131665

[B199] Vollrath M. A. Kwan K. Y. Corey D. P. (2007). The micromachinery of mechanotransduction in hair cells. Annu. Rev. Neurosci. 30, 339–365. 10.1146/annurev.neuro.29.051605.112917 17428178 PMC2865174

[B200] Walker L. M. Publicover S. J. Preston M. R. Said Ahmed M. A. El Haj A. J. (2000). Calcium-channel activation and matrix protein upregulation in bone cells in response to mechanical strain. J. Cell. Biochem. 79, 648–661. 10.1002/1097-4644(20001215)79:4<648:AID-JCB130>3.0.CO;2-Q 10996855

[B201] Wang D. Christensen K. Chawla K. Xiao G. Krebsbach P. H. Franceschi R. T. (1999). Isolation and characterization of MC3T3-E1 preosteoblast subclones with distinct in vitro and in vivo differentiation/mineralization potential. J. Bone Miner. Res. 14, 893–903. 10.1359/jbmr.1999.14.6.893 10352097

[B202] Wang J. H.-C. Grood E. S. Florer J. Wenstrup R. (2000). Alignment and proliferation of MC3T3-E1 osteoblasts in microgrooved silicone substrata subjected to cyclic stretching. J. Biomech. 33, 729–735. 10.1016/S0021-9290(00)00013-0 10807994

[B203] Wang J. H.-C. Jia F. Gilbert T. W. Woo S. L.-Y. (2003). Cell orientation determines the alignment of cell-produced collagenous matrix. J. Biomech. 36, 97–102. 10.1016/S0021-9290(02)00233-6 12485643

[B204] Wang Y. Azaïs T. Robin M. Vallée A. Catania C. Legriel P. (2012). The predominant role of collagen in the nucleation, growth, structure and orientation of bone apatite. Nat. Mater. 11, 724–733. 10.1038/nmat3362 22751179

[B205] Weinbaum S. Cowin S. C. Zeng Y. (1994). A model for the excitation of osteocytes by mechanical loading-induced bone fluid shear stresses. J. Biomech. 27, 339–360. 10.1016/0021-9290(94)90010-8 8051194

[B206] Weiner S. Arad T. Sabanay I. Traub W. Adrian M. Dubochet J. (1997). Rotated plywood structure of primary lamellar bone in the rat: orientations of the collagen fibril arrays. Bone 20, 509–514. 10.1016/S8756-3282(97)00053-7 9177863

[B207] Weinreb M. Rodan G. Thompson D. (1989). Osteopenia in the immobilized rat hind limb is associated with increased bone resorption and decreased bone formation. Bone 10, 187–194. 10.1016/8756-3282(89)90052-5 2803854

[B208] Wenstrup R. J. Florer J. B. Brunskill E. W. Bell S. M. Chervoneva I. Birk D. E. (2004). Type V collagen controls the initiation of collagen fibril assembly. J. Biol. Chem. 279, 53331–53337. 10.1074/jbc.M409622200 15383546

[B209] Westendorf J. J. Kahler R. A. Schroeder T. M. (2004). Wnt signaling in osteoblasts and bone diseases. Gene 341, 19–39. 10.1016/j.gene.2004.06.044 15474285

[B210] Wolff J. (1892). Das Gesetz der Transformation der Knochen (Berlin A. Hirchwild). Translated as: The Law of Bone Remodeling. Berlin: Springer-Verlag.

[B211] Woo S. M. Rosser J. Dusevich V. Kalajzic I. Bonewald L. F. (2011). Cell line IDG-SW3 replicates osteoblast-to-late-osteocyte differentiation in vitro and accelerates bone formation in vivo. J. Bone Miner. Res. 26, 2634–2646. 10.1002/jbmr.465 21735478 PMC3192242

[B212] Xing J. Li Y. Lin M. Wang J. Wu J. Ma Y. (2014). Surface chemistry modulates osteoblasts sensitivity to low fluid shear stress. J. Biomed. Mater. Res. A 102, 4151–4160. 10.1002/jbm.a.35087 24443183

[B213] Xu J. Li Z. Hou Y. Fang W. (2015). Potential mechanisms underlying the Runx2 induced osteogenesis of bone marrow mesenchymal stem cells. Am. J. Transl. Res. 7, 2527–2535. 26885254 PMC4731654

[B214] Yang J.-Y. Ting Y.-C. Lai J.-Y. Liu H.-L. Fang H.-W. Tsai W.-B. (2009). Quantitative analysis of osteoblast-like cells (MG63) morphology on nanogrooved substrata with various groove and ridge dimensions. J. Biomed. Mater. Res. A 90, 629–640. 10.1002/jbm.a.32130 18563818

[B215] Yellowley C. E. Li Z. Zhou Z. Jacobs C. R. Donahue H. J. (2000). Functional gap junctions between osteocytic and osteoblastic cells. J. Bone Miner. Res. 15, 209–217. 10.1359/jbmr.2000.15.2.209 10703922

[B216] You J. Reilly G. C. Zhen X. Yellowley C. E. Chen Q. Donahue H. J. (2001a). Osteopontin gene regulation by oscillatory fluid flow via intracellular calcium mobilization and activation of mitogen-activated protein kinase in MC3T3-E1 osteoblasts. J. Biol. Chem. 276, 13365–13371. 10.1074/jbc.M009846200 11278573

[B217] You L. Cowin S. C. Schaffler M. B. Weinbaum S. (2001b). A model for strain amplification in the actin cytoskeleton of osteocytes due to fluid drag on pericellular matrix. J. Biomech. 34, 1375–1386. 10.1016/S0021-9290(01)00107-5 11672712

[B218] You J. Yellowley C. E. Donahue H. J. Zhang Y. Chen Q. Jacobs C. R. (2000). Substrate deformation levels associated with routine physical activity are less stimulatory to bone cells relative to loading-induced oscillatory fluid flow. J. Biomech. Eng. 122, 387–393. 10.1115/1.1287161 11036562

[B219] You L.-D. Weinbaum S. Cowin S. C. Schaffler M. B. (2004). Ultrastructure of the osteocyte process and its pericellular matrix. Anat. Rec. A Discov. Mol. Cell. Evol. Biol. 278, 505–513. 10.1002/ar.a.20050 15164337

[B220] Young M. F. (2003). Bone matrix proteins: their function, regulation, and relationship to osteoporosis. Osteoporos. Int. 14(Suppl. 3), S35–S42. 10.1007/s00198-002-1342-7 12730768

[B221] Young S. R. L. Hum J. M. Rodenberg E. Turner C. H. Pavalko F. M. (2011). Non-overlapping functions for Pyk2 and FAK in osteoblasts during fluid shear stress-induced mechanotransduction. PLoS ONE 6:e16026. 10.1371/journal.pone.0016026 21283581 PMC3026802

[B222] Yu W. Qu H. Hu G. Zhang Q. Song K. Guan H. (2014). A microfluidic-based multi-shear device for investigating the effects of low fluid-induced stresses on osteoblasts. PLoS ONE 9:e89966. 10.1371/journal.pone.0089966 24587156 PMC3937402

[B223] Zhang K. Barragan-Adjemian C. Ye L. Kotha S. Dallas M. Lu Y. (2006). E11/gp38 selective expression in osteocytes: regulation by mechanical strain and role in dendrite elongation. Mol. Cell. Biol. 26, 4539–4552. 10.1128/MCB.02120-05 16738320 PMC1489126

[B224] Zheng W. Wang Z. Zhang W. Jiang X. (2010). A simple PDMS-based microfluidic channel design that removes bubbles for long-term on-chip culture of mammalian cells. Lab. Chip 10, 2906–2910. 10.1039/c005274d 20844778

[B225] Zhou X. Liu D. You L. Wang L. (2010). Quantifying fluid shear stress in a rocking culture dish. J. Biomech. 43, 1598–1602. 10.1016/j.jbiomech.2009.12.028 20185133 PMC2866761

[B226] Zhu B. Lu Q. Yin J. Hu J. Wang Z. (2005). Alignment of osteoblast-like cells and cell-produced collagen matrix induced by nanogrooves. Tissue Eng. 11, 825–834. 10.1089/ten.2005.11.825 15998222

